# From reads to genes to pathways: differential expression analysis of RNA-Seq experiments using Rsubread and the edgeR quasi-likelihood pipeline

**DOI:** 10.12688/f1000research.8987.2

**Published:** 2016-08-02

**Authors:** Yunshun Chen, Aaron T. L. Lun, Gordon K. Smyth

**Affiliations:** 1 The Walter and Eliza Hall Institute of Medical Research, Parkville, Victoria, 3052, Australia; 2 Department of Medical Biology, The University of Melbourne, Victoria, 3010, Australia; 3 Cancer Research UK Cambridge Institute, University of Cambridge, Li Ka Shing Centre, Cambridge, UK; 4 Department of Mathematics and Statistics, The University of Melbourne, Victoria, 3010, Australia

**Keywords:** RNA sequencing, molecular pathways, gene expression, R software

## Abstract

In recent years, RNA sequencing (RNA-seq) has become a very widely used technology for profiling gene expression. One of the most common aims of RNA-seq profiling is to identify genes or molecular pathways that are differentially expressed (DE) between two or more biological conditions. This article demonstrates a computational workflow for the detection of DE genes and pathways from RNA-seq data by providing a complete analysis of an RNA-seq experiment profiling epithelial cell subsets in the mouse mammary gland. The workflow uses R software packages from the open-source Bioconductor project and covers all steps of the analysis pipeline, including alignment of read sequences, data exploration, differential expression analysis, visualization and pathway analysis. Read alignment and count quantification is conducted using the Rsubread package and the statistical analyses are performed using the edgeR package. The differential expression analysis uses the quasi-likelihood functionality of edgeR.

## Introduction

In recent years, RNA sequencing (RNA-seq) has become a very widely used technology for profiling transcriptional activity in biological systems. One of the most common aims of RNA-seq profiling is to identify genes or molecular pathways that are differentially expressed (DE) between two or more biological conditions. Changes in expression can then be associated with differences in biology, providing avenues for further investigation into potential mechanisms of action.

This article provides a detailed workflow for analyzing an RNA-seq study from the raw reads through to differential expression and pathway analysis using Bioconductor packages ^1^. The article gives a complete analysis of RNA-seq data that were collected to study the effects of pregnancy and lactation on the luminal cell lineage in the mouse mammary gland ^2^. The pipeline uses the *Rsubread* package ^3^ for mapping reads and assigning them to genes, and the *edgeR* package ^4^ for statistical analyses.

RNA-seq analysis involves a number of steps, including read alignment, read summarization, differential expression and pathway analysis. Here we use the Subread aligner ^3^ for mapping and featureCounts ^5^ for assigning reads to genes. As well as being fast and efficient, these algorithms have the advantage of having native implementations as R functions in the *Rsubread* package. This means that the entire analysis can be conducted efficiently within the R environment.

The workflow uses *edgeR*’s quasi-likelihood pipeline (edgeR-quasi) for differential expression. This statistical methodology uses negative binomial generalized linear models ^6^ but with F-tests instead of likelihood ratio tests ^7^. This method provides stricter error rate control than other negative binomial based pipelines, including the traditional *edgeR* pipelines ^6, 8, 9^ or *DESeq2* ^10^. The edgeR-quasi pipeline is based on similar statistical methodology to that of the *QuasiSeq* package ^7^, which has performed well in third-party comparisons ^11^. Compared to *QuasiSeq*, the *edgeR* functions offer speed improvements and some additional statistical refinements ^12^. The RNA-seq pipelines of the *limma* package also offer excellent error rate control ^13, 14^. While the *limma* pipelines are recommended for large-scale datasets, because of their speed and flexibility, the edgeR-quasi pipeline gives better performance in low-count situations ^15, 16^. For the data analyzed here, the edgeR-quasi, limma-voom and limma-trend pipelines are all equally suitable and give similar results.

The analysis approach illustrated in this article can be applied to any RNA-seq study that includes some replication, but it is especially appropriate for designed experiments with multiple treatment factors and with small numbers of biological replicates. The approach assumes that RNA samples have been extracted from cells of interest under two or more treatment conditions, that RNA-seq profiling has been applied to each RNA sample and that there are independent biological replicates for at least one of the treatment conditions. The *Rsubread* part of the workflow takes FASTQ files of raw sequence reads as input, while the *edgeR* part of the pipeline takes a matrix of genewise read counts as input.

## Description of the biological experiment

This workflow demonstrates a complete bioinformatics analysis of an RNA-seq study that is available from the GEO repository as series GSE60450. The RNA-seq data were collected to study the lineage of luminal cells in the mouse mammary gland and in particular how the expression profiles of the members of the lineage change upon pregnancy and lactation ^2^. Specifically, the study examined the expression profiles of basal stem-cell enriched cells (B) and committed luminal cells (L) in the mammary glands of virgin, pregnant and lactating mice. There are therefore six groups of RNA samples, one for each combination of cell type and mouse status. Two biological replicates were collected for each group.

This study used an Illumina Hiseq sequencer to generate about 30 million 100bp single-end reads for each sample. Subread version 1.4.4 ( http://subread.sourceforge.net) was used to align the reads to the mouse mm10 genome and featureCounts was used to assign reads to Entrez Genes using RefSeq gene annotation. The FASTQ files containing the raw sequence reads were deposited to the Sequence Read Archive (SRA) repository and the read counts were deposited to GEO.

This experimental design is summarized in the table below, where the basal and luminal cell types are abbreviated with B and L respectively. The GEO and SRA identifiers for each RNA sample are also shown:



                    > targets 
                    <- 
                    read.delim
                    (
                    "targets.txt"
                    , 
                    stringsAsFactors
                    =
                    FALSE
                    )
> targets

               GEO        SRA CellType    Status
MCL1.DG GSM1480297 SRR1552450        B    virgin
MCL1.DH GSM1480298 SRR1552451        B    virgin
MCL1.DI GSM1480299 SRR1552452        B  pregnant
MCL1.DJ GSM1480300 SRR1552453        B  pregnant
MCL1.DK GSM1480301 SRR1552454        B lactating
MCL1.DL GSM1480302 SRR1552455        B lactating
MCL1.LA GSM1480291 SRR1552444        L    virgin
MCL1.LB GSM1480292 SRR1552445        L    virgin
MCL1.LC GSM1480293 SRR1552446        L  pregnant
MCL1.LD GSM1480294 SRR1552447        L  pregnant
MCL1.LE GSM1480295 SRR1552448        L lactating
MCL1.LF GSM1480296 SRR1552449        L lactating
                


The experiment can be viewed as a one-way layout with six groups. For later use, we combine the treatment factors into a single grouping factor:



                    > group 
                    <- 
                    paste
                    (targets
                    $
                    CellType, targets
                    $
                    Status, 
                    sep
                    =
                    "."
                    )
> group 
                    <- 
                    factor
                    (group)
> 
                    table
                    (group)

group
B.lactating B.pregnant B.virgin L.lactating L.pregnant L.virgin
          2          2        2           2          2        2
                


**Note.** This study isolated carefully sorted cell populations obtained from genetically indentical mice under controlled laboratory conditions. As will be shown during the analysis below, the relatively small sample size ( *n* = 2 in each group) here is justified by the low background variability and by the fact that the expression profiles of the different groups are distinctly different. Studies with more variability (for example on human subjects) or with smaller effect sizes may well require more biological replicates for reliable results.

## Preliminary analysis

### Downloading the read counts

Readers wishing to reproduce the analysis presented in this article can either download the matrix of read counts from GEO or recreate the read count matrix from the raw sequence counts. We will present first the analysis using the downloaded matrix of counts. At the end of this article we will present the R commands needed to recreate this matrix.

The following commands download the genewise read counts for the GEO series GSE60450. The zipped tab-delimited text file GSE60450_Lactation-GenewiseCounts.txt.gz will be downloaded to the working R directory:



                        > FileURL 
                        <- 
                        paste
                        (
+   
                        "
                            http://www.ncbi.nlm.nih.gov/geo/download/?acc=GSE60450"
                        ,
+   
                        "format=file"
                        ,
+   
                        "file=GSE60450_Lactation-GenewiseCounts.txt.gz"
                        ,

                        +   
                        sep
                        =
                        "&"
                        )
> 
                        download.file
                        (FileURL, 
                        "GSE60450_Lactation-GenewiseCounts.txt.gz"
                        )
                    


The counts can then be read into a data.frame in R:



                        > GenewiseCounts 
                        <- 
                        read.delim
                        (
                        "GSE60450_Lactation-GenewiseCounts.txt.gz"
                        ,
+                                 
                        row.names
                        =
                        "EntrezGeneID"
                        )
> 
                        colnames
                        (GenewiseCounts) 
                        <- 
                        substring
                        (
                        colnames
                        (GenewiseCounts),
                        1
                        ,
                        7
                        )
> 
                        dim
                        (GenewiseCounts)

[1] 27179    13

> 
                        head
                        (GenewiseCounts)

          Length MCL1.DG MCL1.DH MCL1.DI MCL1.DJ MCL1.DK MCL1.DL MCL1.LA MCL1.LB
497097      3634     438     300      65     237     354     287       0       0
100503874   3259       1       0       1       1       0       4       0       0
100038431   1634       0       0       0       0       0       0       0       0
19888       9747       1       1       0       0       0       0      10       3
20671       3130     106     182      82     105      43      82      16      25
27395       4203     309     234     337     300     290     270     560     464
          MCL1.LC MCL1.LD MCL1.LE MCL1.LF
497097          0       0       0       0
100503874       0       0       0       0     
100038431       0       0       0       0
19888          10       2       0       0
20671          18       8       3      10
27395         489     328     307     342
                    


The row names of GenewiseCounts are the Entrez Gene Identifiers. The first column contains the length of each gene, being the total number of bases in exons and UTRs for that gene. The remaining 12 columns contain read counts and correspond to rows of targets.

The *edgeR* package stores data in a simple list-based data object called a DGEList. This object is easy to use as it can be manipulated like an ordinary list in R, and it can also be subsetted like a matrix. The main components of a DGEList object are a matrix of read counts, sample information in the data.frame format and optional gene annotation. We enter the counts into a DGEList object using the function DGEList in *edgeR*:



                        > 
                        library
                        (edgeR)
> y 
                        <- 
                        DGEList
                        (GenewiseCounts[,-
                        1
                        ], 
                        group
                        =group,
+               
                        genes
                        =GenewiseCounts[,
                        1
                        ,
                        drop
                        =
                        FALSE
                        ])
> 
                        options
                        (
                        digits
                        =
                        3
                        )
> y
                        $
                        samples

              group lib.size norm.factors
MCL1.DG    B.virgin 23227641            1
MCL1.DH    B.virgin 21777891            1
MCL1.DI  B.pregnant 24100765            1
MCL1.DJ  B.pregnant 22665371            1
MCL1.DK B.lactating 21529331            1
MCL1.DL B.lactating 20015386            1
MCL1.LA    L.virgin 20392113            1
MCL1.LB    L.virgin 21708152            1
MCL1.LC  L.pregnant 22241607            1
MCL1.LD  L.pregnant 21988240            1
MCL1.LE L.lactating 24723827            1
MCL1.LF L.lactating 24657293            1
                    


### Adding gene annotation

The Entrez Gene Ids link to gene information in the NCBI database. The *org.Mm.eg.db* package can be used to complement the gene annotation information. Here, a column of gene symbols is added to y$genes:



                        > 
                        library
                        (org.Mm.eg.db)
> y
                        $
                        genes
                        $
                        Symbol 
                        <- 
                        mapIds
                        (org.Mm.eg.db, 
                        rownames
                        (y),
+                             
                        keytype
                        =
                        "ENTREZID"
                        , 
                        column
                        =
                        "SYMBOL"
                        )
> 
                        head
                        (y
                        $
                        genes)

          Length   Symbol
497097      3634     Xkr4
100503874   3259  Gm19938
100038431   1634  Gm10568
19888       9747      Rp1
20671       3130    Sox17
27395       4203   Mrpl15
                    


Entrez Ids that no longer have official gene symbols are dropped from the analysis. The whole DGEList object, including annotation as well as counts, can be subsetted by rows as if it was a matrix:



                        > y 
                        <- 
                        y[
                        !
                        is.na
                        (y
                        $
                        genes
                        $
                        Symbol), ]
> 
                        dim
                        (y)

[1] 26357    12
                    


### Filtering to remove low counts

Genes that have very low counts across all the libraries should be removed prior to downstream analysis. This is justified on both biological and statistical grounds. From biological point of view, a gene must be expressed at some minimal level before it is likely to be translated into a protein or to be considered biologically important. From a statistical point of view, genes with consistently low counts are very unlikely be assessed as significantly DE because low counts do not provide enough statistical evidence for a reliable judgement to be made. Such genes can therefore be removed from the analysis without any loss of information.

As a rule of thumb, we require that a gene have a count of at least 10–15 in at least some libraries before it is considered to be expressed in the study. We could explicitly select for genes that have at least a couple of counts of 10 or more, but it is slightly better to base the filtering on count-per-million (CPM) values so as to avoid favoring genes that are expressed in larger libraries over those expressed in smaller libraries. For the current analysis, we keep genes that have CPM values above 0.5 in at least two libraries:



                        > keep 
                        <- 
                        rowSums
                        (
                        cpm
                        (
                        y) 
                        > 
                        0.5
                        ) 
                        >= 
                        2

                        > 
                        table
                        (keep)


                        keep
FALSE  TRUE
10704 15653
                    


Here the cutoff of 0.5 for the CPM has been chosen because it is roughly equal to 10 */L* where *L* is the minimum library size in millions. The library sizes here are 20–25 million. We used a round value of 0.5 just for simplicity; the exact value is not important because the downstream differential expression analysis is not sensitive to the small changes in this parameter. The requirement of ≥ 2 libraries is because each group contains two replicates. This ensures that a gene will be retained if it is expressed in both the libraries belonging to any of the six groups.

The above filtering rule attempts to keep the maximum number of interesting genes in the analysis, but other sensible filtering criteria are also possible. For example keep <-rowSums(y$counts) > 50 is a very simple criterion that would keep genes with a total read count of more than 50. This would give similar downstream results for this dataset to the filtering actually used. Whatever the filtering rule, it should be independent of the information in the targets file. It should not make any reference to which RNA libraries belong to which group, because doing so would bias the subsequent differential expression analysis.

The DGEList object is subsetted to retain only the non-filtered genes:



                        > y 
                        <- 
                        y[keep, , 
                        keep.lib.sizes
                        =
                        FALSE
                        ]
                    


The option keep.lib.sizes=FALSE causes the library sizes to be recomputed after the filtering. This is generally recommended, although the effect on the downstream analysis is usually small.

### Normalization for composition bias

Normalization by trimmed mean of M values (TMM) ^17^ is performed by using the calcNormFactors function, which returns the DGEList argument with only the norm.factors changed. It calculates a set of normalization factors, one for each sample, to eliminate composition biases between libraries. The product of these factors and the library sizes defines the effective library size, which replaces the original library size in all downstream analyses.



                        > y 
                        <- 
                        calcNormFactors
                        (y)
> y
                        $
                        samples

              group lib.size norm.factors
MCL1.DG    B.virgin 23139638        1.235
MCL1.DH    B.virgin 21689576        1.213
MCL1.DI  B.pregnant 23976634        1.126
MCL1.DJ  B.pregnant 22546909        1.069
MCL1.DK B.lactating 21422164        1.036
MCL1.DL B.lactating 19918278        1.087
MCL1.LA    L.virgin 20276400        1.370
MCL1.LB    L.virgin 21571124        1.368
MCL1.LC  L.pregnant 22120647        1.006
MCL1.LD  L.pregnant 21879947        0.924
MCL1.LE L.lactating 24660577        0.529
MCL1.LF L.lactating 24602860        0.535
                    


The normalization factors of all the libraries multiply to unity. A normalization factor below one indicates that a small number of high count genes are monopolizing the sequencing, causing the counts for other genes to be lower than would be usual given the library size. As a result, the effective library size will be scaled down for that sample. Here we see that the luminal-lactating samples have low normalization factors. This is a sign that these samples contain a number of very highly upregulated genes.

**Note.** In general, we find TMM normalization to be satisfactory for almost all well-designed mRNA gene expression experiments. Single-cell RNA-seq is an exception, for which specialized normalization methods are needed ^18^. Another, less common, type of study requiring special treatment is that with global differential expression, with more than half of the genome differentially expressed between experimental conditions in the same direction ^19^. Global differential expression should generally be avoided in well designed experiments. When it can’t be avoided, then some normalization reference such as spike-ins needs to be built into the experiment for reliable normalization to be done ^20^.

### Exploring differences between libraries

The RNA samples can be clustered in two dimensions using multi-dimensional scaling (MDS) plots. This is both an analysis step and a quality control step to explore the overall differences between the expression profiles of the different samples. Here we decorate the MDS plot to indicate the cell groups:



                    > pch 
                    <- c
                    (
                    0
                    ,
                    1
                    ,
                    2
                    ,
                    15
                    ,
                    16
                    ,
                    17
                    )

                    > colors 
                    <- rep
                    (
                    c
                    (
                    "darkgreen"
                    , 
                    "red"
                    , 
                    "blue"
                    ), 
                    2
                    )

                    > 
                    plotMDS
                    (y, 
                    col
                    =colors[group], 
                    pch
                    =pch[group])

**Figure 1.**
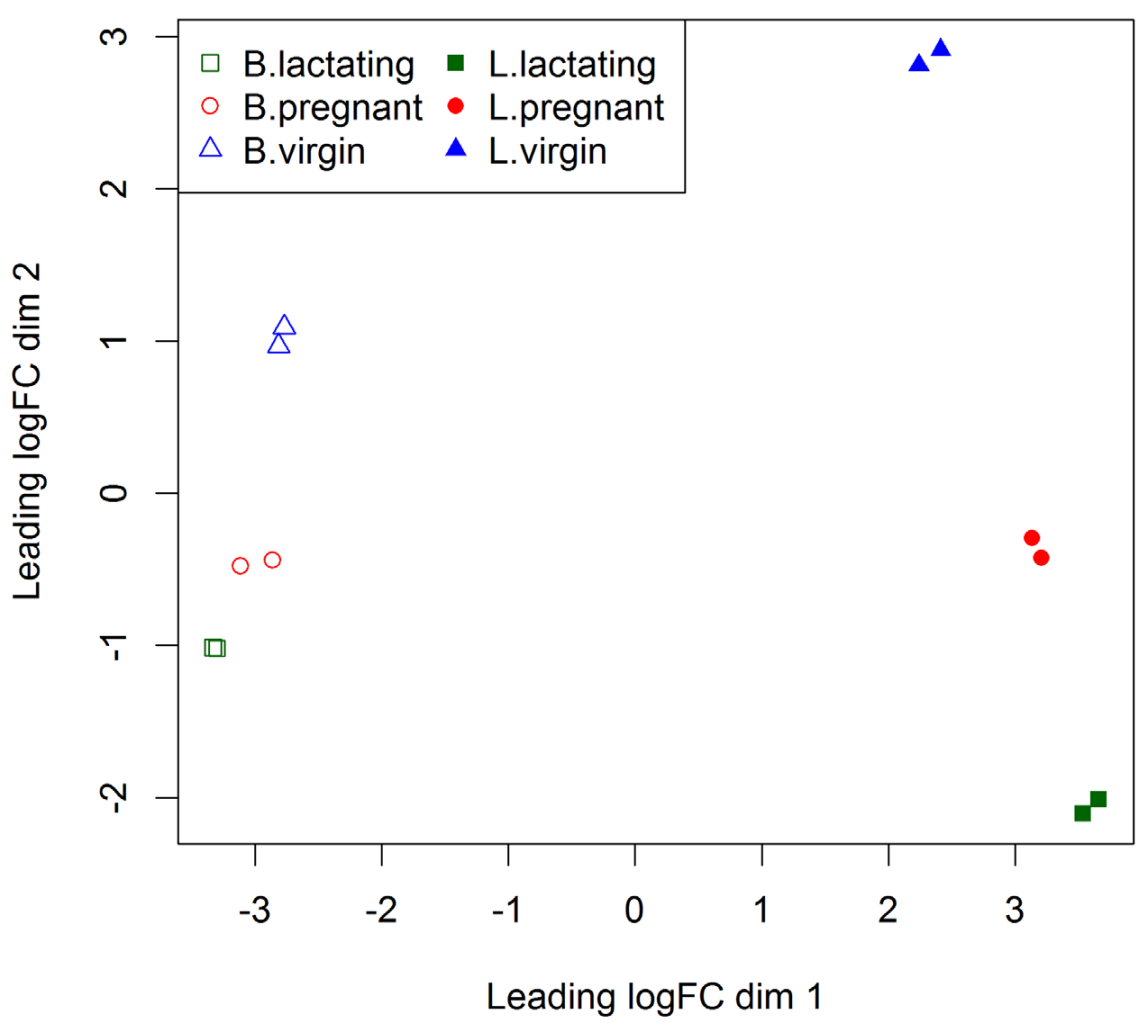
The MDS plot of the data set. Samples are separated by the cell type in the first dimension, and by the mouse status in the second dimension.

                    > 
                    legend
                    (
                    "topleft"
                    , 
                    legend
                    =
                    levels
                    (group), 
                    pch
                    =pch, 
                    col
                    =colors, 
                    ncol
                    =
                    2
                    )


(see Figure 1). In the MDS plot, the distance between each pair of samples can be interpreted as the leading log-fold change between the samples for the genes that best distinguish that pair of samples. By default, leading fold-change is defined as the root-mean-square of the largest 500 log2-fold changes between that pair of samples. Figure 1 shows that replicate samples from the same group cluster together while samples from different groups are well separated. In other words, differences between groups are much larger than those within groups, meaning that there are likely to be statistically significant differences between the groups. The distance between basal cells on the left and luminal cells on the right is about six units on the x-axis, corresponding to a leading fold change of about 64-fold between the two cell types. The differences between the virgin, pregnant and lactating expression profiles appear to be magnified in luminal cells compared to basal.

The expression profiles of individual samples can be explored more closely with mean-difference (MD) plots. An MD plot visualizes the library size-adjusted log-fold change between two libraries (the difference) against the average log-expression across those libraries (the mean). The following command produces an MD plot that compares sample 1 to an artificial reference library constructed from the average of all the other samples:



                    > 
                    plotMD
                    (y, 
                    column
                    =
                    1
                    )

**Figure 2.**
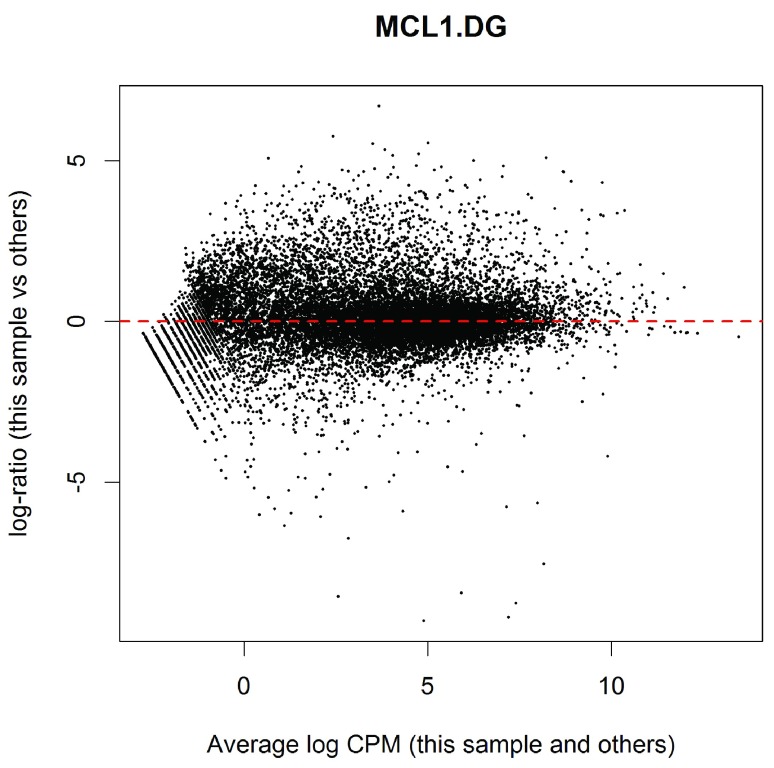
MD plot of log2-expression in sample 1 versus the average log2-expression across all other samples. Each point represents a gene, and the red line indicates a log-ratio of zero. The majority of points cluster around the red line.

                    > 
                    abline
                    (
                    h
                    =
                    0
                    , 
                    col
                    =
                    "red"
                    , 
                    lty
                    =
                    2
                    , 
                    lwd
                    =
                    2
                    )


(see Figure 2). The bulk of the genes are centered around the line of zero log-fold change. The diagonal lines in the lower left of the plot correspond to genes with counts of 0, 1, 2 and so on in the first sample.

It is good practice to make MD plots for all the samples as a quality check. We now look at one of the luminal lactating samples that were observed have low normalization factors:



                    > 
                    plotMD
                    (y, 
                    column
                    =
                    11
                    )

**Figure 3.**
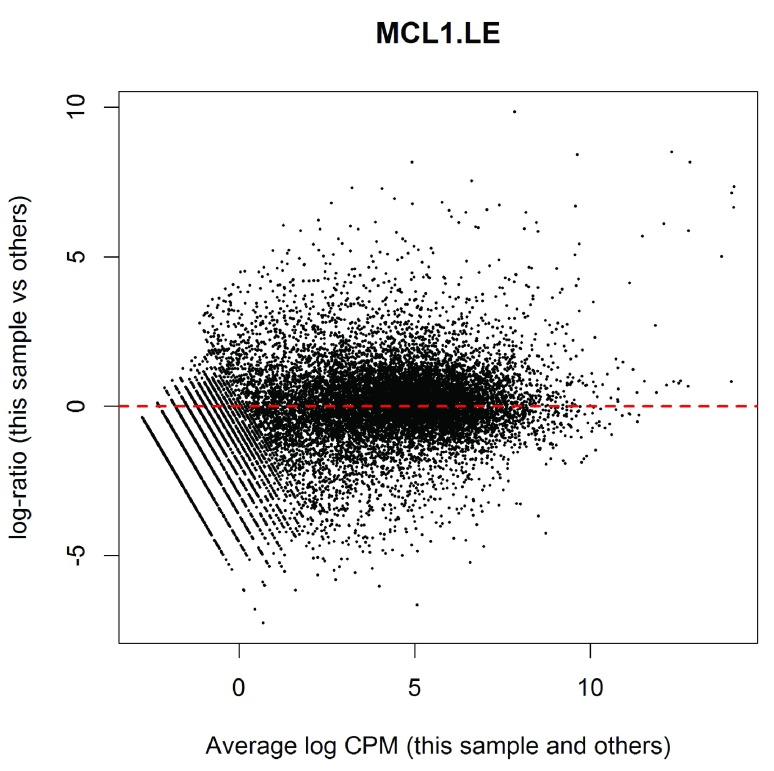
MD plot of log2-expression in sample 11 versus the average log2-expression across all other samples. The plot shows a number of genes that are both highly expressed and highly up-regulated.

                    > 
                    abline
                    (
                    h
                    =
                    0
                    , 
                    col
                    =
                    "red"
                    , 
                    lty
                    =
                    2
                    , 
                    lwd
                    =
                    2
                    )


(see Figure 3). For this sample, the log-ratios show noticeable positive skew, with a number of very highly upregulated genes. In particular, there are a number of points in the upper right of the plot, corresponding to genes that are both highly expressed and highly up-regulated in this sample compared to others. These genes explain why the normalization factor for this sample is well below one. By contrast, the log-ratios for sample 1 were somewhat negatively skewed, corresponding to a normalization factor above one.

### Design matrix

Linear modeling and differential expression analysis in *edgeR* requires a design matrix to be specified. The design matrix records which treatment conditions were applied to each samples, and it also defines how the experimental effects are parametrized in the linear models. The experimental design for this study can be viewed as a one-way layout and the design matrix can be constructed in a simple and intuitive way by:


                    > design 
                    <- 
                    model.matrix
                    (
                    
                        ^~^
                    
                    0
                    +
                    group)
> 
                    colnames
                    (design) 
                    <- 
                    levels
                    (group)
> design

   B.lactating B.pregnant B.virgin L.lactating L.pregnant L.virgin
1            0          0        1           0          0        0
2            0          0        1           0          0        0
3            0          1        0           0          0        0
4            0          1        0           0          0        0
5            1          0        0           0          0        0
6            1          0        0           0          0        0
7            0          0        0           0          0        1
8            0          0        0           0          0        1
9            0          0        0           0          1        0
10           0          0        0           0          1        0
11           0          0        0           1          0        0
12           0          0        0           1          0        0
attr(,"assign")
[1] 1 1 1 1 1 1
attr(,"contrasts")
attr(,"contrasts")$group
[1] "contr.treatment"
                

This design matrix simply links each group to the samples that belong to it. Each row of the design matrix corresponds to a sample whereas each column represents a coefficient corresponding to one of the six groups.

### Dispersion estimation

*edgeR* uses the negative binomial (NB) distribution to model the read counts for each gene in each sample. The dispersion parameter of the NB distribution accounts for variability between biological replicates ^6^. *edgeR* estimates an empirical Bayes moderated dispersion for each individual gene. It also estimates a common dispersion, which is a global dispersion estimate averaged over all genes, and a trended dispersion where the dispersion of a gene is predicted from its abundance. Dispersion estimates are most easily obtained from the estimateDisp function:



                        > y 
                        <- 
                        estimateDisp
                        (y, design, 
                        robust
                        =
                        TRUE
                        )
                    


This returns a DGEList object with additional components ( common.dispersion, trended.dispersion and tagwise.dispersion) added to hold the estimated dispersions. Here robust=TRUE has been used to protect the empirical Bayes estimates against the possibility of outlier genes with exceptionally large or small individual dispersions ^21^.

**Figure 4.**
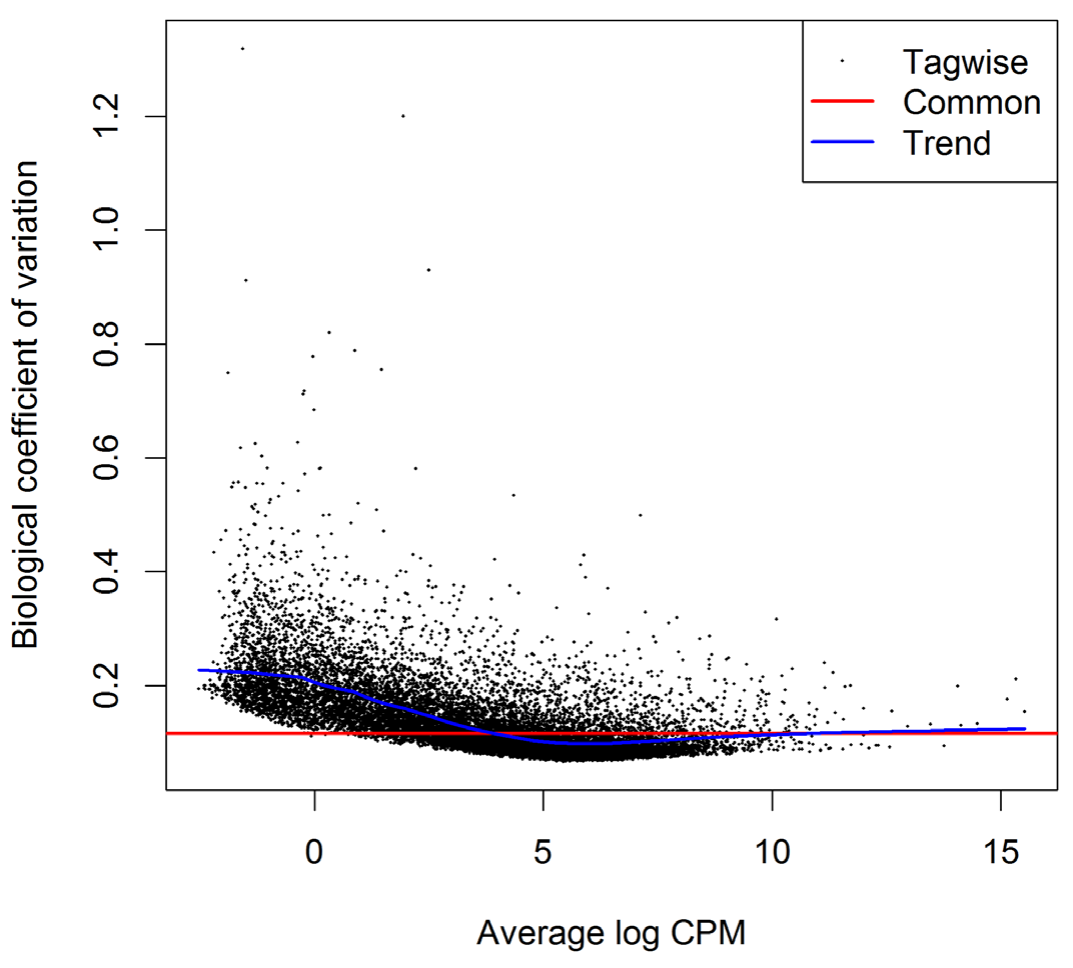
Scatterplot of the biological coefficient of variation (BCV) against the average abundance of each gene. The plot shows the square-root estimates of the common, trended and tagwise NB dispersions.

The dispersion estimates can be visualized with plotBCV:


                    > 
                    plotBCV
                    (y)
                

(see Figure 4). The vertical axis of the plotBCV plot shows square-root dispersion, also known as *biological coefficient of variation* (BCV) ^6^.

For RNA-seq studies, the NB dispersions tend to be higher for genes with very low counts. The dispersion trend tends to decrease smoothly with abundance and to asymptotic to a constant value for genes with larger counts. From our past experience, the asymptotic value for the BCV tends to be in range from 0.05 to 0.2 for genetically identical mice or cell lines, whereas somewhat larger values ( *>* 0.3) are observed for human subjects.

The NB model can be extended with quasi-likelihood (QL) methods to account for gene-specific variability from both biological and technical sources ^7, 12^. Under the QL framework, the NB dispersion trend is used to describe the overall biological variability across all genes, and gene-specific variability above and below the overall level is picked up by the QL dispersion. In the QL approach, the individual (tagwise) NB dispersions are not used.

The estimation of QL dispersions is performed using the glmQLFit function:


                    > fit 
                    <- 
                    glmQLFit
                    (y, design, 
                    robust
                    =
                    TRUE
                    )
> 
                    head
                    (fit
                    $
                    coefficients)

       B.lactating B.pregnant B.virgin L.lactating L.pregnant L.virgin
497097      -11.13     -12.01   -11.22       -19.0     -19.03    -19.0
20671       -12.76     -12.51   -12.15       -14.5     -14.30    -14.1
27395       -11.27     -11.29   -11.53       -10.6     -10.86    -10.9
18777       -10.15     -10.21   -10.76       -10.1     -10.38    -10.4
21399        -9.89      -9.73    -9.78       -10.2      -9.97    -10.0
58175       -16.15     -14.85   -15.98       -13.3     -12.29    -12.0
                

This returns a DGEGLM object with the estimated values of the GLM coefficients for each gene. It also contains a number of empirical Bayes (EB) statistics including the QL dispersion trend, the squeezed QL dispersion estimates and the prior degrees of freedom (df). The QL dispersions can be visualized by plotQLDisp:


                    > 
                    plotQLDisp
                    (fit)
                

(see Figure 5).

**Figure 5.**
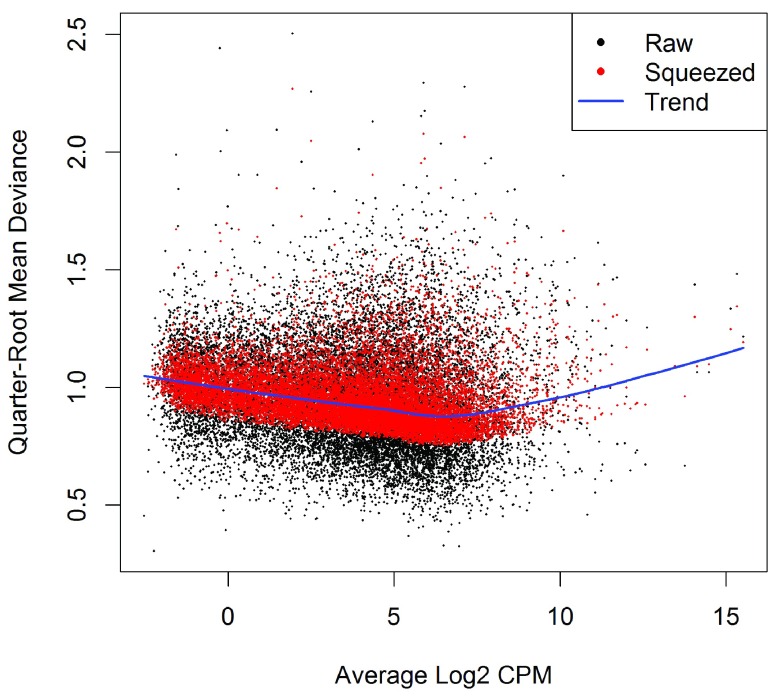
A plot of the quarter-root QL dispersion against the average abundance of each gene. Estimates are shown for the raw (before EB moderation), trended and squeezed (after EB moderation) dispersions. Note that the QL dispersions and trend shown here are relative to the NB dispersion trend shown in Figure 4.

The QL functions moderate the genewise QL dispersion estimates in the same way that the *limma* package moderates variances ^22^. The raw QL dispersion estimates are squeezed towards a global trend, and this moderation reduces the uncertainty of the estimates and improves testing power. The extent of the squeezing is governed by the value of the prior df estimated from the data. Large prior df estimates indicate that the QL dispersions are less variable between genes, meaning that strong EB moderation should be performed. Smaller prior df estimates indicate that the true unknown dispersions are highly variable, so weaker moderation towards the trend is appropriate.


                    > 
                    summary
                    (fit
                    $
                    df.prior)

   Min. 1st Qu. Median Mean 3rd Qu. Max.
   3.00    6.77   6.77 6.63    6.77 6.77
                

Setting robust=TRUE in glmQLFit is usually recommended ^21^. This allows gene-specific prior df estimates, with lower values for outlier genes and higher values for the main body of genes. This reduces the Chance of getting false positives from genes with extremely high or low raw dispersions, while at the same time increasing statistical power to detect differential expression for the main body of genes.

## Differential expression analysis

### Testing for differential expression

The next step is to test for differential expression between the experimental groups. One of the most interesting comparisons is that between the basal pregnant and lactating groups. The contrast corresponding to any specified comparison can be constructed conveniently using the makeContrasts function:


                    > B.LvsP 
                    <- 
                    makeContrasts
                    (B.lactating
                    -
                    B.pregnant, 
                    levels
                    =design)
                

In subsequent results, a positive log _2_-fold-change (logFC) will indicate a gene up-regulated in lactating mice relative to pregnant, whereas a negative logFC will indicate a gene more highly expressed in pregnant mice. We will use QL F-tests instead of the more usual likelihood ratio tests (LRT) as they give stricter error rate control by accounting for the uncertainty in dispersion estimation:


                    > res 
                    <- 
                    glmQLFTest
                    (fit, 
                    contrast
                    =B.LvsP)
                

The top DE genes can be viewed with topTags:


                    > 
                    topTags
                    (res)

Coefficient:   1*B.lactating -1*B.pregnant
       Length   Symbol logFC logCPM   F   PValue      FDR
12992     765  Csn1s2b  6.08  10.19 421 4.78e-11 7.48e-07
211577   2006   Mrgprf  5.15   2.75 343 1.32e-10 7.99e-07
226101   7094     Myof  2.32   6.45 322 1.97e-10 7.99e-07
381290   8292   Atp2b4  2.14   6.15 320 2.04e-10 7.99e-07
140474  11281     Muc4 -7.17   6.06 308 2.60e-10 8.15e-07
231830   3346  Micall2 -2.25   5.19 282 4.41e-10 1.15e-06
24117    2242     Wif1 -1.82   6.77 261 7.13e-10 1.60e-06
12740    1812    Cldn4 -5.32   9.87 298 9.12e-10 1.74e-06
21953     667    Tnni2  5.75   3.86 313 1.00e-09 1.74e-06
231991   2873    Creb5  2.57   4.87 240 1.17e-09 1.83e-06
                

In order to control the false discovery rate (FDR), multiple testing correction is performed using the Benjamini-Hochberg method. The top DE gene *Csn1s2b* has a large positive logFC, showing that it is far more highly expressed in the basal cells of lactating than pregnant mice. This gene is indeed known to be a major source of protein in milk.

The total number of DE genes identified at an FDR of 5% can be shown with decideTestsDGE. There are in fact more than 5000 DE genes in this comparison:


                    > is.de 
                    <- 
                    decideTestsDGE
                    (res)
> 
                    summary
                    (is.de)

   [,1]
-1  2757
0  10408
1   2488
                

**Figure 6.**
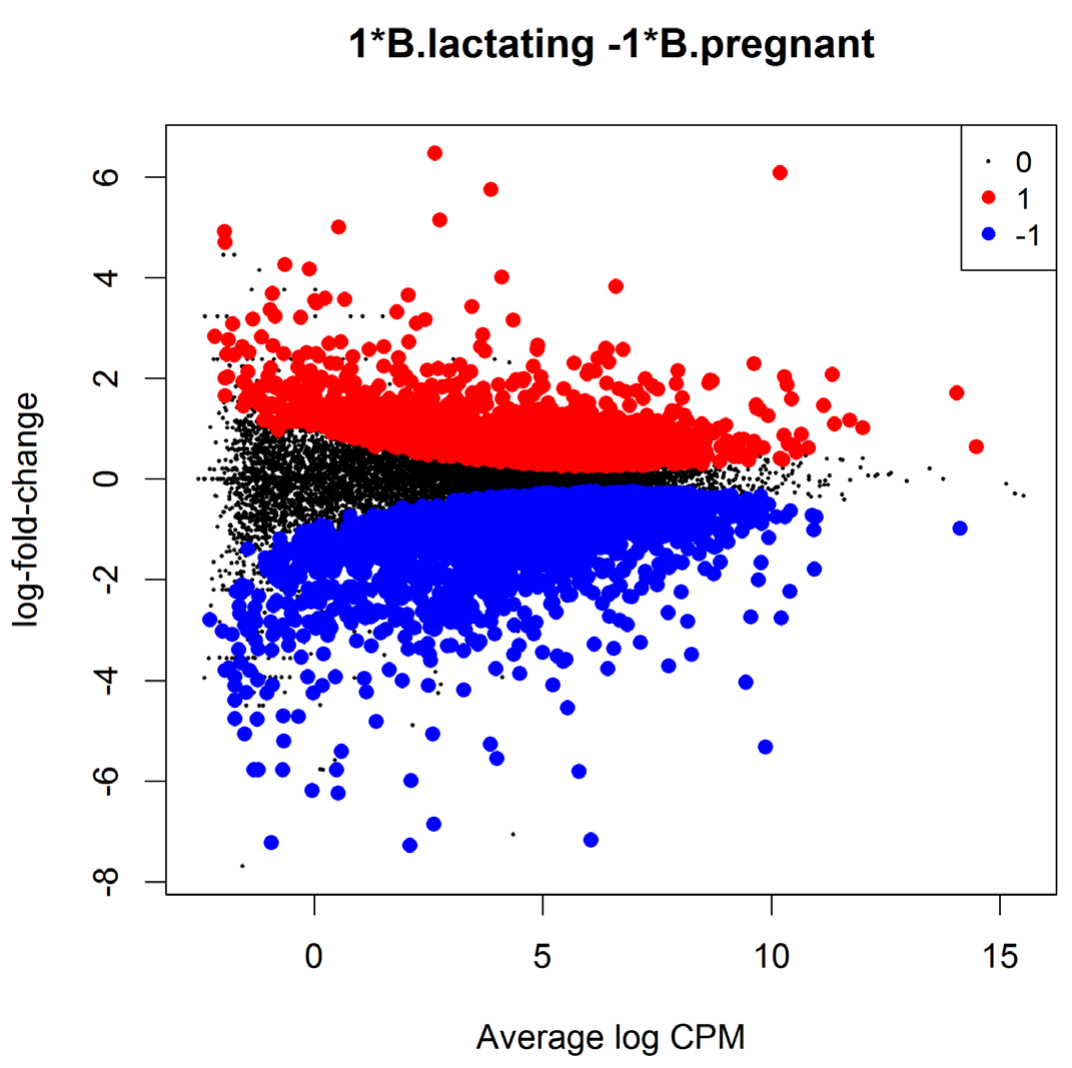
MD plot showing the log-fold change and average abundance of each gene. Significantly up and down DE genes are highlighted in red and blue, respectively.

The magnitude of the differential expression changes can be visualized with a fitted model MD plot:


                    > 
                    plotMD
                    (res, 
                    status
                    =is.de, 
                    values
                    =
                    c
                    (
                    1
                    ,
                    -
                    1
                    ), 
                    col
                    =
                    c
                    (
                    "red"
                    ,
                    "blue"
                    ),

                    +         
                    legend
                    =
                    "topright"
                    )
                

(see Figure 6). The logFC for each gene is plotted against the average abundance in log2-CPM, i.e., logCPM in the table above. Genes that are significantly DE are highlighted:

### Differential expression relative to a fold-change threshold

glmQLFTest identifies differential expression based on statistical significance regardless of how small the difference might be. For some purposes we might be interested only in genes with reasonably large expression changes. The above analysis found more than 5000 DE genes between the basal pregnant and lactating groups. With such a large number of DE genes, it makes sense to narrow down the list to genes that are more biologically meaningful.

A commonly used approach is to apply FDR and logFC cutoffs simultaneously. However this tends to favor lowly expressed genes, and also fails to control the FDR correctly. A better and more rigorous approach is to modify the statistical test so as to detect expression changes greater than a specified threshold. In *edgeR*, this can be done using the glmTreat function. This function is analogous to the TREAT method for microarrays ^23^ but is adapted to the NB framework. Here we test whether the differential expression fold changes are significantly greater than 1.5, that is, whether the logFCs are significantly greater than log _2_(1.5):


                    > tr 
                    <- 
                    glmTreat
                    (fit, 
                    contrast
                    =B.LvsP, 
                    lfc
                    =
                    log2
                    (
                    1.5
                    ))
> 
                    topTags
                    (tr)

Coefficient:   1*B.lactating -1*B.pregnant
       Length   Symbol logFC unshrunk.logFC logCPM   PValue      FDR
12992     765  Csn1s2b  6.08           6.09  10.19 5.94e-11 9.30e-07
211577   2006   Mrgprf  5.15           5.15   2.75 1.77e-10 1.38e-06
140474  11281     Muc4 -7.17          -7.34   6.06 3.98e-10 2.07e-06
226101   7094     Myof  2.32           2.32   6.45 7.82e-10 2.69e-06
381290   8292   Atp2b4  2.14           2.14   6.15 1.01e-09 2.69e-06
12740    1812    Cldn4 -5.32          -5.32   9.87 1.14e-09 2.69e-06
21953     667    Tnni2  5.75           5.76   3.86 1.20e-09 2.69e-06
231830   3346  Micall2 -2.25          -2.25   5.19 1.90e-09 3.71e-06
231991   2873    Creb5  2.57           2.57   4.87 3.55e-09 6.18e-06
16012    1289   Igfbp6  2.87           2.87   3.68 4.60e-09 7.20e-06
                

Note that the argument lfc is an abbreviation for “log-fold-change”. About 1100 genes are detected as DE with a FC significantly above 1.5 at an FDR cut-off of 5%.


                    > is.de 
                    <- 
                    decideTestsDGE
                    (tr)
> 
                    summary
                    (is.de)

   [,1]
-1   723
0  14530
1    400
                

The p-values from glmTreat are larger than those from glmQLFTest, and the number of significantly DE genes is fewer, because it is testing an interval null hypothesis and requires stronger evidence for differential expression than does a conventional test. It provides greater specificity for identifying the most important genes with large fold changes.

**Figure 7.**
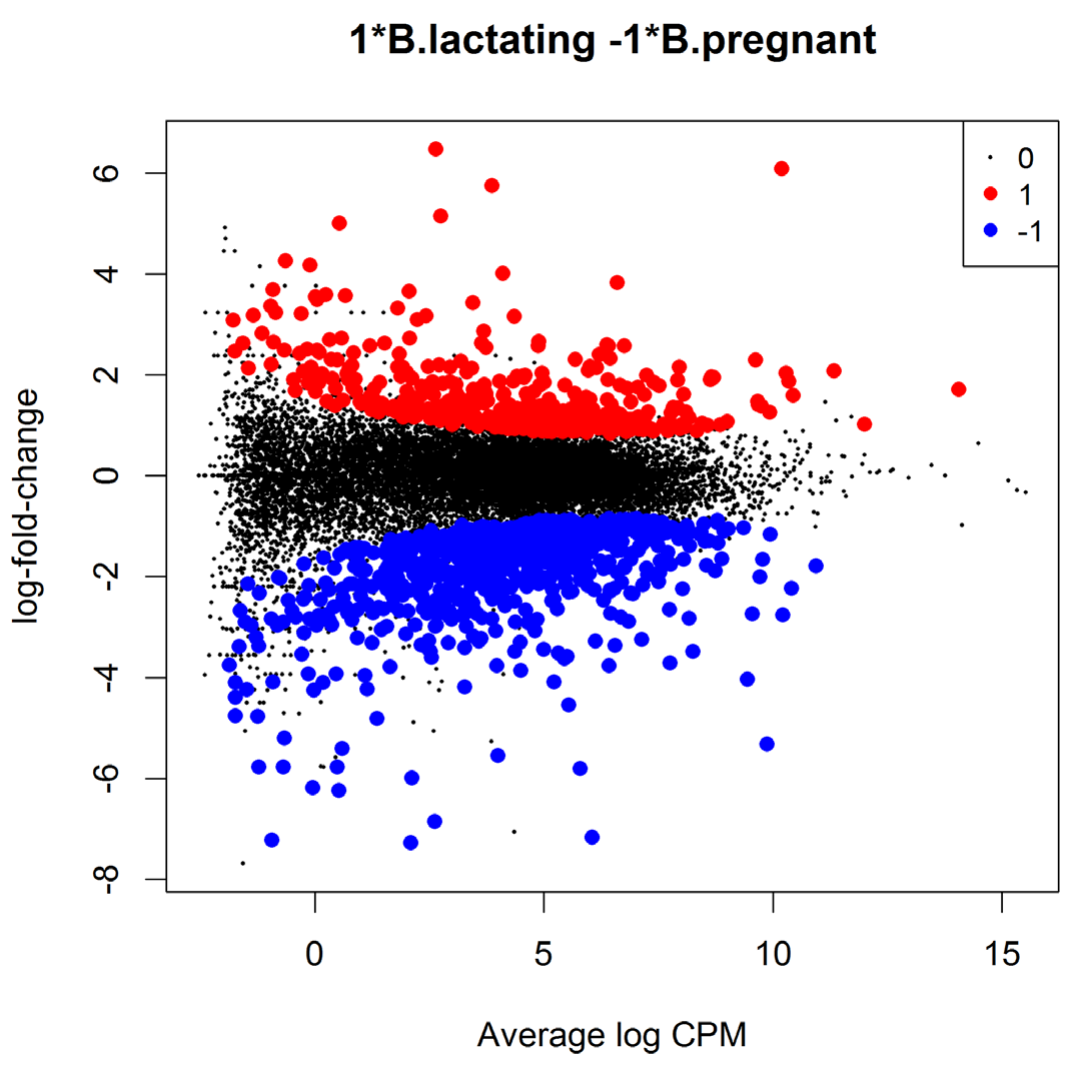
MD plot showing the log-fold change and average abundance of each gene. Genes with fold-changes significantly greater than 1.5 are highlighted.

The test results can be visualized in an MD plot:


                    > 
                    plotMD
                    (tr, 
                    status
                    =is.de, 
                    values
                    =
                    c
                    (
                    1
                    ,
                    -
                    1
                    ), 
                    col
                    =
                    c
                    (
                    "red"
                    ,
                    "blue"
                    ),
+         
                    legend
                    =
                    "topright"
                    )
                

(see Figure 7). The glmTreat method evaluates variability as well as the magnitude of change of expression values and therefore is not equivalent to a simple fold change cutoff. Nevertheless, all the statistically significant expression changes have logFC greater than 0.8 and almost all (97%) are greater than 0.9. These values compare to the threshold value of log _2_(1.5) = 0.58. In general, an estimated logFC must exceed the TREAT threshold by a number of standard errors for it to be called significant. In other words, the whole confidence interval for the logFC must clear the threshold rather than just the estimated value itself. It is better to interpret the threshold as *the FC below which we are definitely not interested in the gene* rather than *the FC above which we are interested in the gene*.

The value of the FC threshold can be varied depending on the dataset. In the presence of a huge number of DE genes, a relatively large FC threshold may be appropriate to narrow down the search to genes of interest. In the absence of DE genes, on the other hand, a small or even no FC threshold shall be used. If the threshold level is set to zero, then glmTreat becomes equivalent to glmQLFTest in the workflow shown here.

In general, using glmTreat to reduce the number of DE genes is better than simply reducing the FDR cutoff, because glmTreat prioritizes genes with larger changes that are likely to be more biologically significant. glmTreat can also be used with *edgeR* pipelines other than quasi-likelihood, although we don’t demonstrate that here.

### Heat map clustering

Heatmaps are a popular way to display differential expression results for publication purposes. To create a heatmap, we first convert the read counts into log2-counts-per-million (logCPM) values. This can be done with the cpm function:


                    > logCPM 
                    <- 
                    cpm
                    (y, 
                    prior.count
                    =
                    2
                    , 
                    log
                    =
                    TRUE
                    )
> 
                    rownames
                    (logCPM) 
                    <- 
                    y
                    $
                    genes
                    $
                    Symbol 
> 
                    colnames
                    (logCPM) 
                    <- 
                    paste
                    (y
                    $
                    samples
                    $
                    group, 
                    1
                    :
                    2
                    , 
                    sep
                    =
                    "-"
                    )
                

The introduction of prior.count is to avoid undefined values and to reduce the variability of the logCPM values for genes with low counts. Larger values for prior.count shrink the logFCs for low count genes towards zero.

We will create a heatmap to visualize the top 30 DE genes according to the TREAT test between B.lactating and B.pregnant. The advantage of a heatmap is that it can display the expression pattern of the genes across all the samples. Visualization of the results is aided by clustering together genes that have correlated expression patterns. First we select the logCPM values for the 30 top genes:


                    > o 
                    <- 
                    order
                    (tr
                    $
                    table
                    $
                    PValue)
> logCPM 
                    <- 
                    logCPM[o[
                    1
                    :
                    30
                    ],]
                

Then we scale each row (each gene) to have mean zero and standard deviation one:


                    > logCPM 
                    <- 
                    t
                    (
                    scale
                    (
                    t
                    (logCPM)))
                

This scaling is commonly done for heatmaps and ensures that the heatmap displays relative changes for each gene. A heat map can then be produced by the heatmap.2 function in the *gplots* package:


                    > 
                    library
                    (gplots)
> col.pan 
                    <- 
                    colorpanel
                    (
                    100
                    , 
                    "blue"
                    , 
                    "white"
                    , 
                    "red"
                    )

**Figure 8.**
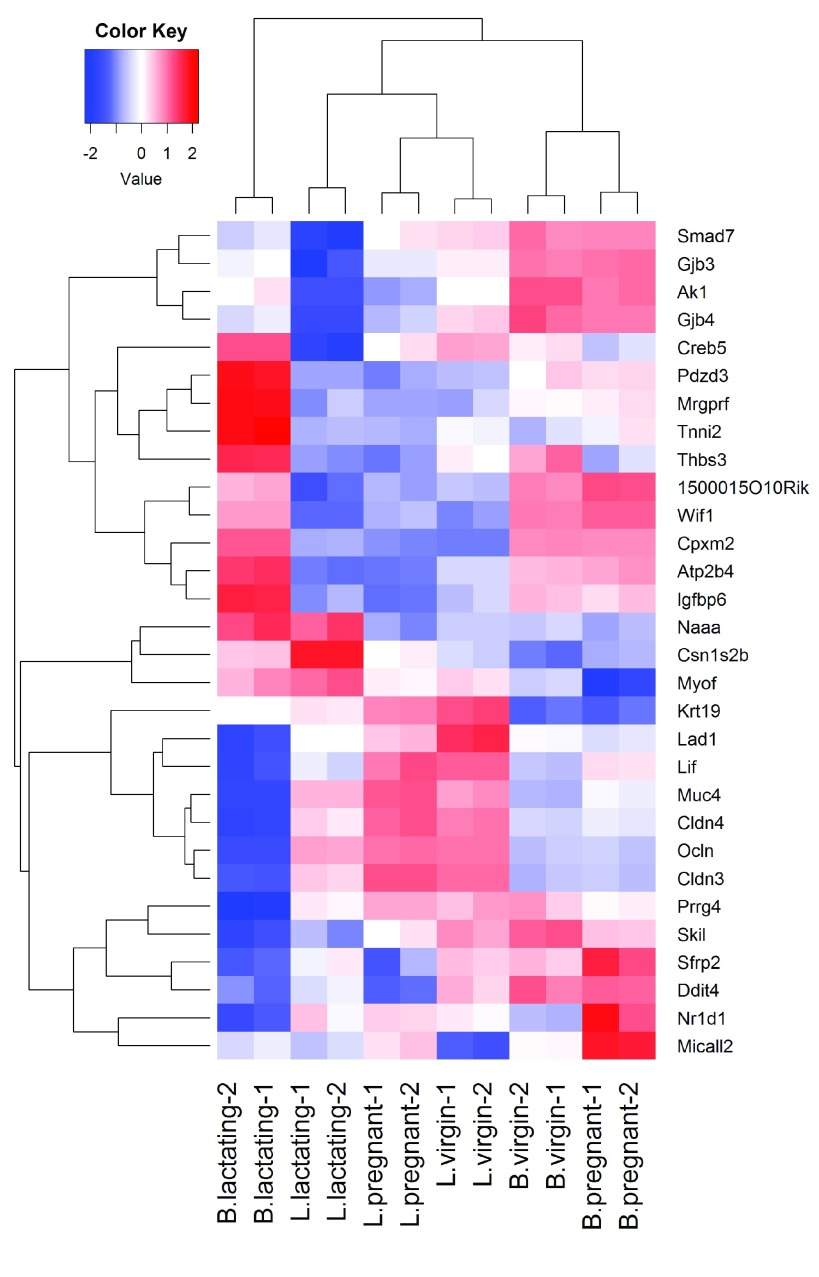
Heat map across all the samples using the top 30 most DE genes between the basal lactating group and the basal pregnancy group.

                    > 
                    heatmap.2
                    (logCPM, 
                    col
                    =col.pan, 
                    Rowv
                    =
                    TRUE
                    , 
                    scale
                    =
                    "none"
                    ,

                    +     
                    trace
                    =
                    "none"
                    , 
                    dendrogram
                    =
                    "both"
                    , 
                    cexRow
                    =
                    1
                    , 
                    cexCol
                    =
                    1.4
                    , 
                    density.info
                    =
                    "none"
                    ,

                    +     
                    margin
                    =
                    c
                    (
                    10
                    ,
                    9
                    ), 
                    lhei
                    =
                    c
                    (
                    2
                    ,
                    10
                    ), 
                    lwid
                    =
                    c
                    (
                    2
                    ,
                    6
                    ))
                

(see Figure 8). By default, heatmap.2 clusters genes and samples based on Euclidean distance between the expression values. Because we have pre-standardized the rows of the logCPM matrix, the Euclidean distance between each pair of genes is proportional to (1 − *r*) ^2^, where *r* is the Pearson correlation coefficient between the two genes. This shows that the heatmap will cluster together genes that have positively correlated logCPM values, because large positive correlations correspond to small distances.

The positioning of the samples in the heatmap is dependent on how the genes in the display have been chosen. Here we are displaying those genes that are most DE between B.lactating and B.pregnant, so those two cell populations are well separated on the plot. As expected, the two replicate samples from each group are clustered together.

### Analysis of deviance

The differential expression analysis comparing two groups can be easily extended to comparisons between three or more groups. This is done by creating a matrix of independent contrasts. In this manner, users can perform a one-way analysis of deviance (ANODEV) for each gene ^24^.

Suppose we want to compare the three groups in the luminal population, i.e., virgin, pregnant and lactating. An appropriate contrast matrix can be created as shown below, to make pairwise comparisons between all three groups:


                    > con 
                    <- 
                    makeContrasts
                    (
+      
                    L.PvsL 
                    = L.pregnant 
                    - 
                    L.lactating,
+      
                    L.VvsL 
                    = L.virgin 
                    - 
                    L.lactating,
+      
                    L.VvsP 
                    = L.virgin 
                    - 
                    L.pregnant, 
                    levels
                    =design)
                

The QL F-test is then applied to identify genes that are DE between the three groups. This combines the three pairwise comparisons into a single F-statistic and p-value. The top set of significant genes can be displayed with topTags:


                    > res 
                    <- 
                    glmQLFTest
                    (fit, 
                    contrast
                    =con)
> 
                    topTags
                    (res)

Coefficient:  LR test of 2 contrasts
      Length  Symbol logFC.L.PvsL logFC.L.VvsL logCPM     F   PValue      FDR
19242   2021     Ptn        -1.54         7.26   7.97  2386 3.73e-17 5.84e-13
13645   4757     Egf        -5.36        -7.22   3.67  1124 4.47e-15 3.15e-11
52150   4089   Kcnk6        -2.42        -7.00   5.91  1020 8.27e-15 3.15e-11
15439   1345      Hp         1.08         5.42   4.93   992 9.89e-15 3.15e-11
12992    765 Csn1s2b        -8.55       -11.36  10.19  1050 1.01e-14 3.15e-11
14183   5346   Fgfr2        -1.15         3.95   7.38   953 1.28e-14 3.17e-11
20856   1793    Stc2        -1.81         3.20   6.11   919 1.60e-14 3.17e-11
11941   7050  Atp2b2        -7.37       -10.56   6.60  1134 1.78e-14 3.17e-11
13358   1678 Slc25a1        -4.13        -4.91   7.50   888 1.99e-14 3.17e-11
17068    691    Ly6d         3.42         9.24   4.69   886 2.02e-14 3.17e-11
                

Note that the three contrasts of pairwise comparisons are linearly dependent. Constructing the contrast matrix with any two of the contrasts would be sufficient for an ANODEV test. If the contrast matrix contains all three possible pairwise comparisons, then only the log-fold changes of the first two contrasts are shown in the output of topTags.

### Complicated contrasts

The flexibility of the GLM framework makes it possible to specify arbitrary contrasts for differential expression tests. Suppose we are interested in testing whether the change in expression between lactating and pregnant mice is the same for basal cells as it is for luminal cells. In statistical terminology, this is the interaction effect between mouse status and cell type. The contrast corresponding to this testing hypothesis can be made as follows.


                    > con 
                    <- 
                    makeContrasts
                    (
+      (L.lactating
                    -
                    L.pregnant)
                    -
                    (B.lactating
                    -
                    B.pregnant),
+       
                    levels
                    =design)
                

Then the QL F-test is conducted to identify genes that are DE under this contrast. The top set of DE genes are viewed with topTags.


                    > res 
                    <- 
                    glmQLFTest
                    (fit, 
                    contrast
                    =con)
> 
                    topTags
                    (res)

Coefficient: -1*B.lactating  1*B.pregnant 1*L.lactating -1*L.pregnant
       Length   Symbol logFC logCPM   F   PValue      FDR
19041    6277      Ppl -4.62   6.96 524 9.55e-12 1.49e-07
231991   2873    Creb5 -5.61   4.87 438 2.91e-11 2.13e-07
20512    4206   Slc1a3  5.03   3.65 415 4.09e-11 2.13e-07
217294   1952 BC006965 -3.88   4.68 372 8.11e-11 2.86e-07
14598    2022     Ggt1  3.17   6.38 357 1.04e-10 2.86e-07
13358    1678  Slc25a1  3.47   7.50 354 1.10e-10 2.86e-07
192166   4558    Sardh  2.92   5.11 342 1.36e-10 3.04e-07
19659    2628     Rbp1 -4.40   6.83 337 1.63e-10 3.18e-07
67547    3707  Slc39a8  6.19   5.07 376 1.83e-10 3.18e-07
14063    2768    F2rl1 -3.92   5.61 302 2.93e-10 4.20e-07
                

## Pathway analysis

### Gene ontology analysis

We now consider the problem of interpreting the differential expression results in terms of higher order biological processes or molecular pathways. One of the most common used resources is gene ontology (GO) databases, which annotate genes according to a dictionary of annotation terms. A simple and often effective way to interpret the list of DE genes is to count the number of DE genes that are annotated with each possible GO term. GO terms that occur frequently in the list of DE genes are said to be over-represented or enriched. In *edgeR*, GO analyses can be conveniently conducted using the goana function. Here were apply goana to the output of the TREAT analysis comparing B.lactating to B.pregnant. The top most significantly enriched GO terms can be viewed with topGO.


                    > go 
                    <- 
                    goana
                    (tr, 
                    species
                    =
                    "Mm"
                    )
> 
                    topGO
                    (go, 
                    n
                    =
                    15
                    )

                                     Term Ont    N Up Down  P.Up   P.Down
GO:0022402             cell cycle process  BP  931 19  118 0.913 2.69e-23
GO:0000280               nuclear division  BP  460 10   78 0.789 3.58e-23
GO:1903047     mitotic cell cycle process  BP  628  8   92 0.995 1.56e-22
GO:0048285              organelle fission  BP  500 11   78 0.785 7.35e-21
GO:0007067       mitotic nuclear division  BP  376  4   66 0.991 1.51e-20
GO:0007049                     cell cycle  BP 1301 21  138 0.997 6.22e-20
GO:0000278             mitotic cell cycle  BP  736  8   96 0.999 7.97e-20
GO:0007059         chromosome segregation  BP  237  1   49 0.998 1.50e-18
GO:0051301                  cell division  BP  550  6   77 0.997 8.95e-18
GO:0000776                    kinetochore  CC  112  1   33 0.952 8.99e-18
GO:0000775 chromosome, centromeric region  CC  163  1   38 0.988 1.77e-16
GO:0098813 nuclear chromosome segregation  BP  171  1   38 0.990 9.64e-16
GO:0042254            ribosome biogenesis  BP  223  1   42 0.998 1.43e-14
GO:0098687             chromosomal region  CC  278  6   47 0.756 2.69e-14
GO:0005730                      nucleolus  CC  663  4   78 1.000 9.72e-14
                

The goana function automatically extracts DE genes from the tr object, and conducts overlap tests for the up- and down-regulated DE genes separately. By default, an FDR cutoff of 5% is used when extracting DE genes, but this can be varied. The row names of the output are the universal identifiers of the GO terms and the Term column gives the human-readable names of the terms. The Ont column shows the ontology domain that each GO term belongs to. The three domains are: biological process (BP), cellular component (CC) and molecular function (MF). The N column represents the total number of genes annotated with each GO term. The Up and Down columns indicate the number of genes within the GO term that are significantly up- and down-regulated in this differential expression comparison, respectively. The P.Up and P.Down columns contain the p-values for over-representation of the GO term in the up- and down-regulated genes, respectively. Note that the p-values are not adjusted for multiple testing—we would usually ignore GO terms with p-values greater than about 10 ^−5^.

By default the output table from topGO is sorted by the minimum of P.Up and P.Down. Other options are available. For example, topGO(go, sort="up") lists the top GO terms that are over-represented in the up-regulated genes. The domain of the enriched GO terms can also be specified by users. For example, topGO(go, ontology="BP") restricts to the top GO terms belonging to the biological process domain while topGO(go, ontology="MF") restricts to molecular function terms.

The goana function uses the NCBI RefSeq annotation and requires the use of Entrez Gene Identifiers.

### KEGG pathway analysis

Another popular annotation database is the Kyoto Encyclopedia of Genes and Genomes (KEGG). Much smaller than GO, this is a curated database of molecular pathways and disease signatures. A KEGG analysis can be done exactly as for GO, but using the kegga function:


                    > keg 
                    <- 
                    kegga
                    (tr, 
                    species
                    =
                    "Mm"
                    )

                    > 
                    topKEGG
                    (keg, 
                    n
                    =
                    15
                    , 
                    truncate
                    =
                    34
                    )
			
					 Pathway    N Up Down     P.Up   P.Down
path:mmu03008 Ribosome biogenesis in eukaryot...   75  1   19 4.04e-01 3.23e-19
path:mmu01100                 Metabolic pathways 1030 28   51 3.42e-10 1.11e-15
path:mmu04110                         Cell cycle  120  1   19 5.63e-01 3.78e-15
path:mmu00230                  Purine metabolism  152  2   18 2.80e-01 3.45e-12
path:mmu00240              Pyrimidine metabolism   96  0   15 1.00e+00 3.86e-12
path:mmu05150    Staphylococcus aureus infection   30  0   10 1.00e+00 4.78e-12
path:mmu04510                     Focal adhesion  191 13    9 7.22e-10 1.34e-03
path:mmu04060 Cytokine-cytokine receptor inte...  167  9   16 2.34e-06 1.35e-09
path:mmu04514     Cell adhesion molecules (CAMs)  112  2   13 1.79e-01 4.72e-09
path:mmu04972               Pancreatic secretion   63  8    4 1.14e-08 1.10e-02
path:mmu04970                 Salivary secretion   63  8    7 1.14e-08 2.45e-05
path:mmu04610 Complement and coagulation casc...   47  2    9 4.14e-02 1.32e-08
path:mmu05166                   HTLV-I infection  245  5   17 2.71e-02 5.39e-08
path:mmu00100               Steroid biosynthesis   18  5    0 1.13e-07 1.00e+00
path:mmu04114                     Oocyte meiosis  101  2   11 1.53e-01 1.43e-07
                

The output from topKEGG is the same as from topGO except that row names become KEGG pathway IDs, Term becomes Pathway and there is no Ont column. Both the GO and KEGG analyses show that the cell cycle pathway is strongly down-regulated upon lactation in mammary stem cells.

By default, the kegga function automatically reads the latest KEGG annotation from the Internet each time it is run. The KEGG database uses Entrez Gene Ids, and the kegga function assumes these are available as the row names of tr.

### FRY gene set tests

The GO and KEGG analyses shown above are relatively simple analyses that rely on a list of DE genes. The list of DE genes is overlapped with the various GO and KEGG annotation terms. The results will depend on the significance threshold that is used to assess differential expression.

If the aim is to test for particular gene expression signatures or particular pathways, a more nuanced approach is to conduct a roast or fry gene set test ^25^. These functions test whether a set of genes is DE, assessing the whole set of genes as a whole. Gene set tests consider all the genes in the specified set and do not depend on any pre-emptive significance cutoff. The set of genes can be chosen to be representative of any pathway or phenotype of interest.

roast gives p-values using random rotations of the residual space. In the *edgeR* context, fry is generally recommended over roast.fry gives an accurate analytic approximation to the results that roast would give, with default settings, if an extremely large number of rotations was used.

Here, suppose we are interested in three GO terms related to cytokinesis. Each GO term is used to define a set of genes annotated with that term. The names of these terms are shown below:



                    > 
                    library
                    (GO.db)

                    > cyt.go 
                    <- 
                    c
                    (
                    "GO:0032465"
                    , 
                    "GO:0000281"
                    , 
                    "GO:0000920"
                    )

                    > term 
                    <- 
                    select
                    (GO.db, 
                    keys
                    =cyt.go, 
                    columns
                    =
                    "TERM"
                    )

                    > term

        GOID                              TERM
1 GO:0032465         regulation of cytokinesis
2 GO:0000281               mitotic cytokinesis
3 GO:0000920 cell separation after cytokinesis
                

The first step is to extract the genes associated with each GO term from the GO database. This produces a list of three components, one for each GO term. Each component is a vector of Entrez Gene IDs for that GO term:


                    > 
                    Rkeys
                    (org.Mm.egGO2ALLEGS) 
                    <- 
                    cyt.go

                    > cyt.go.genes 
                    <- 
                    as.list
                    (org.Mm.egGO2ALLEGS)
                

Suppose the comparison of interest is between the virgin and lactating groups in the basal population. We can use fry to test whether the cytokinesis GO terms are DE for this comparison:


                    > B.VvsL 
                    <- 
                    makeContrasts
                    (B.virgin-B.lactating, 
                    levels
                    =design)

                    > 
                    fry
                    (y, 
                    index
                    =cyt.go.genes, 
                    design
                    =design, 
                    contrast
                    =B.VvsL)

                    
           NGenes Direction   PValue     FDR PValue.Mixed FDR.Mixed
GO:0032465     48        Up 0.000464 0.00139     3.71e-06  7.02e-06
GO:0000920     16      Down 0.001902 0.00285     4.68e-06  7.02e-06
GO:0000281     30        Up 0.007277 0.00728     2.50e-05  2.50e-05
                

Each row of the output corresponds to a gene set. The NGenes column provides the number of genes in each set. The Direction column indicates the net direction of change. The PValue column gives the two-sided p-value for testing whether the set is DE as a whole, either up or down. The PValue.Mixed column gives a p-value for testing whether genes in the set tend to be DE, without regard to direction. The PValue column is appropriate when genes in the set are expected to be co-regulated, all or most changing expression in the same direction. The PValue.Mixed column is appropriate when genes in the set are not necessarily co-regulated or may be regulated in different directions for the contrast in question. FDRs are calculated from the corresponding p-values across all sets.

The results of a gene set test can be viewed in a barcode plot produced by the barcodeplot function. Suppose visualization is performed for the gene set defined by the GO term GO:0032465:


                    > res 
                    <- 
                    glmQLFTest
                    (fit, 
                    contrast
                    =B.VvsL)

**Figure 9.**
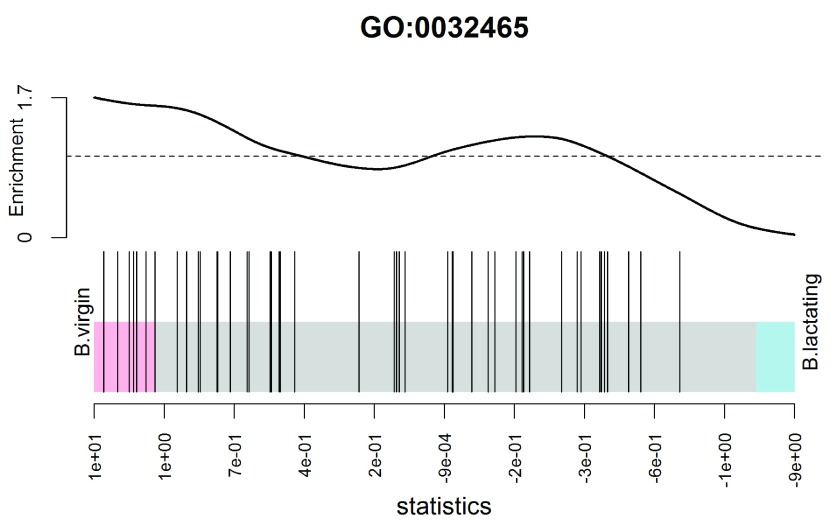
Barcode plot showing enrichment of the GO term GO:0032465 in the basal virgin group compared to the basal lactating group. X-axis shows logFC for B.virgin vs B.lactating. Black bars represent genes annotated with the GO term. The worm shows relative enrichment.

                    > index 
                    <- 
                    rownames
                    (fit) 
                    %in% 
                    cyt.go.genes[[
                    1
                    ]]
> 
                    barcodeplot
                    (res
                    $
                    table
                    $
                    logFC, 
                    index
                    =index, 
                    labels
                    =
                    c
                    (
                    "B.virgin"
                    ,
                    "B.lactating"
                    ),
+               
                    main
                    =cyt.go[
                    1
                    ])
                

(see Figure 9). In the plot, all genes are ranked from left to right by decreasing log-fold change for the contrast and the genes within the gene set are represented by vertical bars, forming the barcode-like pattern. The curve (or *worm*) above the barcode shows the relative local enrichment of the bars in each part of the plot. The dotted horizontal line indicates neutral enrichment; the worm above the dotted line shows enrichment while the worm below the dotted line shows depletion. In this particular barcode plot the worm shows enrichment on the left for positive logFCs, and depletion on the right for negative logFCs. The conclusion is that genes associated with this GO term tend to be up-regulated in the basal cells of virgin mice compared to lactating mice, confirming the result of the fry test above.

### Camera gene set enrichment analysis

Finally we demonstrate a gene set enrichment style analysis using the Molecular Signatures Database (MSigDB) ^26^. We will use the C2 collection of the MSigDB, which is a collection of nearly 5000 curated gene sets, each representing the molecular signature of a particular biological process or phenotype. The MSigDB itself is purely human, but the Walter and Eliza Hall Institute (WEHI) maintains a mouse version of the database. We load the mouse version of the C2 collection from the WEHI website:


                    > 
                    load
                    (
                    url
                    (
                    "
                        http://bioinf.wehi.edu.au/software/MSigDB/mouse_c2_v5p1.rdata"
                    ))
                

This will load Mm.c2, which is a list of gene sets, each a vector of Entrez Ids. This can be converted to a list of index numbers:


                    > idx 
                    <- 
                    ids2indices
                    (Mm.c2,
                    id
                    =
                    rownames
                    (y))
                

First we compare virgin stem cells to virgin luminal cells:


                    > BvsL.v 
                    <- 
                    makeContrasts
                    (B.virgin - L.virgin, 
                    levels
                    =design)

                    > cam 
                    <- 
                    camera
                    (y, idx, design, 
                    contrast
                    =BvsL.v, 
                    inter.gene.cor
                    =
                    0.01
                    )

                    > 
                    options
                    (
                    digits
                    =
                    2
                    )

                    > 
                    head
                    (cam,
                    14
                    )
                                                    NGenes Direction  PValue
LIM_MAMMARY_STEM_CELL_UP                               782        Up 2.0e-43
LIM_MAMMARY_LUMINAL_MATURE_DN                          169        Up 3.7e-25
SASAI_RESISTANCE_TO_NEOPLASTIC_TRANSFROMATION           80        Up 4.1e-20
LIM_MAMMARY_STEM_CELL_DN                               664      Down 2.2e-19
FARMER_BREAST_CANCER_CLUSTER_4                          74        Up 2.9e-19
NABA_BASEMENT_MEMBRANES                                 52        Up 1.6e-17
HAEGERSTRAND_RESPONSE_TO_IMATINIB                       35        Up 4.0e-17
ROZANOV_MMP14_TARGETS_SUBSET                            83        Up 5.2e-16
REACTOME_COLLAGEN_FORMATION                             70        Up 1.3e-15
REACTOME_NCAM1_INTERACTIONS                             71        Up 2.2e-15
OXFORD_RALB_TARGETS_UP                                  27        Up 4.5e-15
ANASTASSIOU_CANCER_MESENCHYMAL_TRANSITION_SIGNATURE    148        Up 7.1e-15
PAPASPYRIDONOS_UNSTABLE_ATEROSCLEROTIC_PLAQUE_DN        68        Up 1.3e-14
LIM_MAMMARY_LUMINAL_PROGENITOR_UP                       92      Down 2.0e-14
                                                        FDR
LIM_MAMMARY_STEM_CELL_UP                            9.3e-40
LIM_MAMMARY_LUMINAL_MATURE_DN                       8.9e-22
SASAI_RESISTANCE_TO_NEOPLASTIC_TRANSFROMATION       6.4e-17
LIM_MAMMARY_STEM_CELL_DN                            2.7e-16
FARMER_BREAST_CANCER_CLUSTER_4                      2.8e-16
NABA_BASEMENT_MEMBRANES                             1.2e-14
HAEGERSTRAND_RESPONSE_TO_IMATINIB                   2.7e-14
ROZANOV_MMP14_TARGETS_SUBSET                        3.0e-13
REACTOME_COLLAGEN_FORMATION                         6.8e-13
REACTOME_NCAM1_INTERACTIONS                         1.0e-12
OXFORD_RALB_TARGETS_UP                              1.9e-12
ANASTASSIOU_CANCER_MESENCHYMAL_TRANSITION_SIGNATURE 2.8e-12
PAPASPYRIDONOS_UNSTABLE_ATEROSCLEROTIC_PLAQUE_DN    4.9e-12
LIM_MAMMARY_LUMINAL_PROGENITOR_UP                   6.8e-12
                

With a large gene set collection, setting inter.gene.cor = 0.01 gives a good compromise between biological interpretability and FDR control. As expected, the mammary stem cell and mammary luminal cell signatures from Lim *et al.* ^27^ are top-ranked, and in the expected directions.

We can visualize the top signature, combining the up and down mammary stem cell signatures to make a bi-directional signature set:


                    > res 
                    <- 
                    glmQLFTest
                    (fit, 
                    contrast
                    =BvsL.v)
> 
                    barcodeplot
                    (res
                    $
                    table
                    $
                    logFC,
+               
                    index
                    =idx[[
                    "LIM_MAMMARY_STEM_CELL_UP"
                    ]],
+               
                    index2
                    =idx[[
                    "LIM_MAMMARY_STEM_CELL_DN"
                    ]],
+               
                    labels
                    =
                    c
                    (
                    "B.virgin"
                    ,
                    "L.virgin"
                    ),
+               
                    main
                    =
                    "LIM_MAMMARY_STEM_CELL"
                    ,
+               
                    alpha
                    =
                    1
                    ,)
                

(see Figure 10).

**Figure 10.**
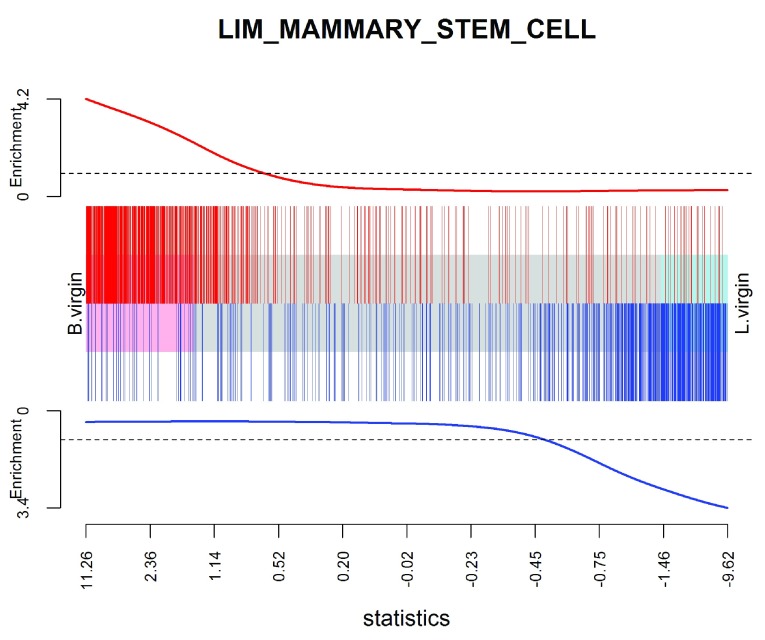
Barcode plot showing strong enrichment of mammary stem cell signature in the stem cell vs luminal cell comparison. Red bars show up signature genes, blue bars show down genes. The worms show relative enrichment.

## Packages used

This workflow depends on various packages from version 3.3 of the Bioconductor project, running on R version 3.3.0 or higher. The complete list of the packages used for this workflow are shown below:


                > 
                sessionInfo
                ()

R version 3.3.1 (2016-06-21)
Platform: x86_64-w64-mingw32/x64 (64-bit)
Running under: Windows 7 x64 (build 7601) Service Pack 1

locale:
[1] LC_COLLATE=English_Australia.1252    LC_CTYPE=English_Australia.1252
[3] LC_MONETARY=English_Australia.1252   LC_NUMERIC=C
[5] LC_TIME=English_Australia.1252

attached base packages:
[1] parallel  stats4   stats   graphics  grDevices  utils    datasets  methods
[9] base

other attached packages:
 [1] GO.db_3.3.0          gplots_3.0.1        org.Mm.eg.db_3.3.0
 [4] AnnotationDbi_1.34.3 IRanges_2.6.0       S4Vectors_0.10.2
 [7] Biobase_2.32.0       BiocGenerics_0.18.0 edgeR_3.14.0
[10] limma_3.28.6         BiocStyle_2.0.2     knitr_1.13

loaded via a namespace (and not attached):
 [1] magrittr_1.5       splines_3.3.1      statmod_1.4.24   lattice_0.20-33
 [5] stringr_1.0.0      highr_0.6          caTools_1.17.1   tools_3.3.1
 [9] grid_3.3.1         KernSmooth_2.23-15 DBI_0.4-1        gtools_3.5.0
[13] formatR_1.4        bitops_1.0-6       evaluate_0.9     RSQLite_1.0.0
[17] gdata_2.17.0       stringi_1.1.1      locfit_1.5-9.1
            

## Read alignment and quantification

### Download raw sequence files from the SRA

We now revisit the question of recreating the matrix of read counts from the raw sequence reads. Unlike the above workflow, which works for any version of R, read alignment requires Unix or Mac OS and, in practice, a high performance Unix server is recommended. Read alignment and read counting require only one Bioconductor package, *Rsubread*. However the fastq-dump utility from the SRA Toolkit is also required to convert from SRA to FASTQ format. This can be downloaded from the NCBI website ( http://www.ncbi.nlm.nih.gov/Traces/sra/?view=software) and installed on any Unix system.

The first task is to download the raw sequence files, which are stored in SRA format on the SRA repository. The SRA files need to be unpacked into FASTQ format using the fastq-dump utility. The following R code makes a system call to fastq-dump to download each SRA file and convert it to FASTQ format:


                    > for (sra in targets$SRA) system(paste("fastq-dump", sra))
                

The fastq-dump utility automatically downloads the specified SRA data set from the internet. The above code will produce 12 FASTQ files, in the current working directory, with file names given by the following vector:


                    > all.fastq <- paste0(targets$SRA, ".fastq")
                

### Accuracy of base-calling

Sequencers typically store base-calling quality scores for each read in the FASTQ files. *Rsubread*’s qualityScores function can be used to extract these scores from any particular file:



                    > QS <- qualityScores("SRR1552444.fastq")


**Figure 11.**
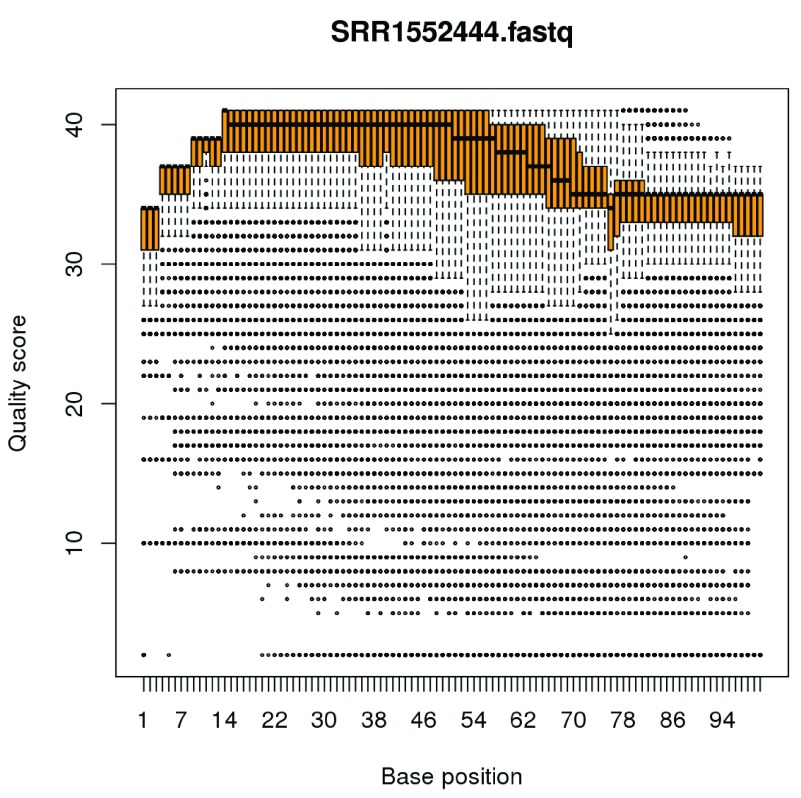
Boxplots of quality scores by base position for the first FASTQ file.

The boxplot function provides a compact way to view the quality scores by position across all reads:



                    
> boxplot(QS, ylab="Quality score", xlab="Base position",
+         main="SRR1552444.fastq", cex=0.25, col="orange")

                

(see Figure 11). The vertical axis shows the Phred quality score, equal to *−*10 log _10_ ( *p*) where *p* is the probability of an erroneous call. The maximum possible value is 40, and all values above 10 correspond to extremely small error probabilities. The horizontal axis shows position within a read. The file contains 100bp single-end reads, so the scale is from 1 to 100. The plot displays a compact boxplot at each base position. As is very commonly observed, the quality scores are best in the middle of the reads and decrease slightly towards the start and end of the reads. However the quality remains generally good even near the ends of the reads: the scores would need to be very much lower than this before they would cause problems for the alignment. Similar plots can be made for each of the FASTQ files.

### Build a genome index

Before the sequence reads can be aligned, we need to build an index for the GRCm38/mm10 (Dec 2011) build of the mouse genome. Most laboratories that use *Rsubread* regularly will already have an index file prepared, as this is a once-off operation for each genome release. If you are using *Rsubread* for mouse for the first time, then the latest mouse genome build can be downloaded from the NCBI location ftp://ftp.ncbi.nlm.nih.gov/genomes/all/GCA_000001635.6_GRCm38.p4/GCA_000001635.6_GRCm38.p4_genomic.fna.gz. (Note that this link is for patch 4 of mm10, which is valid at the time of writing in May 2016. The link will change as new patches are released periodically.) An index can then be built by:


                    > library(Rsubread)
> buildindex(basename = "mm10",
+            reference = "GCA_000001635.6_GRCm38.p4_genomic.fna.gz")
                

### Aligning reads

The sequence reads can now be aligned to the mouse genome using the align function:


                    > all.bam <- sub(".fastq", ".bam", all.fastq)
> align(index="mm10", readfile1=all.fastq, input_format="FASTQ",
+       output_file=all.bam)
                

This produces a set of BAM files containing the read alignments for each RNA library. The mapping proportions can be summarized by the propmapped function:


                    > propmapped(all.bam)
          Samples NumTotal NumMapped PropMapped
1  SRR1552450.bam 30109290  26577308      0.883
2  SRR1552451.bam 28322351  24794251      0.875
3  SRR1552452.bam 31688348  27937620      0.882
4  SRR1552453.bam 29614284  26074034      0.880
5  SRR1552454.bam 27225012  24381742      0.896
6  SRR1552455.bam 25433157  22813815      0.897
7  SRR1552444.bam 27919481  23927833      0.857
8  SRR1552445.bam 29731031  25487822      0.857
9  SRR1552446.bam 29879070  25500318      0.853
10 SRR1552447.bam 29245388  25187577      0.861
11 SRR1552448.bam 31425424  27326500      0.870
12 SRR1552449.bam 31276061  27204156      0.870
                

Ideally, the proportion of mapped reads should be above 80%. By default, only reads with unique mapping locations are reported by *Rsubread* as being successfully mapped. Restricting to uniquely mapped reads is recommended, as it avoids spurious signal from non-uniquely mapped reads derived from, e.g., repeat regions.

### Quantifying read counts for each gene

The read counts for each gene can be quantified using the featureCounts function in *Rsubread*. Conveniently, the *Rsubread* package includes inbuilt NCBI RefSeq annotation of the mouse and human genomes. featureCounts generates a matrix of read counts for each gene in each sample:


                    > fc <- featureCounts(all.bam, annot.inbuilt="mm10")
                

The output is a simple list, containing the matrix of counts ( counts), a data frame of gene characteristics ( annotation), a vector of file names ( targets) and summary mapping statistics ( stat):


                    > names(fc)
[1] "counts"		"annotation"	"targets"  "stat"
                

The row names of fc$counts are the Entrez gene identifiers for each gene. The column names are the output file names from align, which we simplify here for brevity:


                    > colnames(fc$counts) <- rownames(targets)
                

The first six rows of the counts matrix are shown below.


                    > head(fc$counts)
          MCL1.DG MCL1.DH MCL1.DI MCL1.DJ MCL1.DK MCL1.DL MCL1.LA MCL1.LB MCL1.LC
497097        438     299      65     237     354     287       0       0       0
100503874       1       0       1       1       0       4       0       0       0
100038431       0       0       0       0       0       0       0       0       0
100038431       0       0       0       0       0       0       0       0       0
19888           1       1       0       0       0       0      10       3      10
20671         106     181      82     104      43      83      16      25      18
27395         309     232     339     290     291     270     558     468     488
          MCL1.LD MCL1.LE MCL1.LF
497097          0       0       0
100503874       0       0       0
100038431       0       0       0
19888           2       0       0
20671           8       3      10
27395         332     312     344
                

Finally, a DGEList object can be assembled by:


                    > y <- DGEList(fc$counts, group=group)
> y$genes <- fc$annotation[, "Length", drop=FALSE]
                

## Data and software availability

Except for the targets file targets.txt, all data analyzed in the workflow is read automatically from public websites as part of the code. All software used is publicly available as part of Bioconductor 3.3, except for the fastq-dump utility, which can be downloaded from NCBI website as described in the text. The article includes the complete code necessary to reproduce the analyses shown.

The LaTeX version of this article was generated automatically by running knitr::knit on an Rnw file of R commands. It is planned to make the code and data available as an executable Bioconductor workflow at http://www.bioconductor.org/help/workflows. In the meantime, the files are available from http://bioinf.wehi.edu.au/edgeR/F1000Research2016/.
