# Supplementary material for: From reads to genes to pathways: differential expression analysis of RNA-Seq experiments using Rsubread and the edgeR quasi-likelihood pipeline
Source: F1000Res. 2016 Aug 2;5:1438. Originally published 2016 Jun 20. [Version 2] doi: 10.12688/f1000research.8987.2 (PMC4934518; doi:10.12688/f1000research.8987.2)
Supplement: Peer review history

# Peer review: Referee response for version 2

**DOI:** 10.5256/f1000research.9996.r15361

**Published:** 2016-08-08

**Authors:** Conrad J. Burden

**Affiliations:** 1 Mathematical Sciences Institute, Australian National University, Canberra, ACT, Australia; 2 Research School of Biology, Australian National University, Canberra, ACT, Australia

I have read this submission. I believe that I have an appropriate level of expertise to confirm that it is of an acceptable scientific standard.

# Peer review: Referee response for version 2

**DOI:** 10.5256/f1000research.9996.r15360

**Published:** 2016-08-02

**Authors:** Nicholas J. Schurch

**Affiliations:** 1 Division of Computational Biology, College of Life Sciences, University of Dundee, Dundee, UK

I have read this submission. I believe that I have an appropriate level of expertise to confirm that it is of an acceptable scientific standard.

# Peer review: Referee response for version 2

**DOI:** 10.5256/f1000research.9996.r15362

**Published:** 2016-08-02

**Authors:** Devon P. Ryan

**Affiliations:** 1 Max Planck Institute of Immunobiology and Epigenetics, Freiburg, Germany

I have read this submission. I believe that I have an appropriate level of expertise to confirm that it is of an acceptable scientific standard.

# Peer review: Referee response for version 1

**DOI:** 10.5256/f1000research.9667.r14474

**Published:** 2016-07-18

**Authors:** Steve Lianoglou

**Affiliations:** 1 Department of Bioinformatics and Computational Biology, Genentech Inc., San Francisco, CA, USA

**General Comments**

 I'd like to first thank the authors for having a long history of providing key contributions to the filed of bioinformatics in terms of methodological advances which are manifested in robust software (limma and edgeR), and further for being most generous with their time by providing extensive support for their software and its use by writing (epic) user guides and answering an innumerably many questions on the bioconductor support forum -- especially since the latter is likely not considered "important" (citable(!)) under most models of academic recognition. The community owes you a large debt of gratitude.

 Now, for this workflow: The authors have provided a complete tutorial on the analysis of RNA-seq data that addresses many of the considerations required while performing these tasks. I'm particularly happy to see that the authors draw people's attention to the use of their "treat" framework, the brief (but important) ANODEV section, as well as discussing different ways to perform gene set enrichment analysis.

 My strongest comment is that this is very well written and should prove very useful to the community at large -- and most useful to the "casual" analyst, one who isn't well versed in the various avenues of research that are now so conveniently wrapped up behind a single call to `glmTreat` or `camera`. In this vein, I feel the authors have done a commendable job of touching upon many of the more subtle parts of the data preparation steps in an RNA-seq analysis (ie. the importance of filtering, how normalization factors are used to adjust library size, explanation of an MDS plot, etc).

 Of course one can't comment on every corner case that might arise during an rna-seq analysis, but to benefit this audience most, I'd like to point out some points that I feel could benefit from further clarification. Other readers would likely wish the authors clarify another set of points. The set of points that are most important to discuss is going to be subjective and based on our own experience in analyzing datasets (and helping others do the same), but for me I feel at least these could use some more elaboration:

1. After the `plotMD` code example, the authors mention that "the bulk of genes should be centered at a line of zero log-fold change ...", it might be worth mentioning a few options to consider when a vanilla call to `calcNormFactors` doesn't produce that result.
2. It is ultimately the user's responsibility to keep up with the primary publications in the field, but I think the authors can help with just a few clarifying comments.

- In the Introduction, the authors cite [12] (A. Lun, et al. It's DE-licious ...) when they mention edgeR's QLF framework offers some additional statistical refinements when compared to QuasiSeq, but [12] doesn't seem to mention any direct comparisons to QuasiSeq at all. As far as I can tell, [12] only mentions that QuasiSeq also uses quasi-likelihood F-tests, and that these account for gene-specific variability from both biological and technical sources. Could the authors clarify what these refinements might be? (In retrospect, this comment seems a bit trivial to make. I intended to chase more comments to publications, but unfortunately don't have the time ... I doubt that there's any need to, just trying to help the casual analyst connect some dots here, is all)
- From previous experience of putting camera's `inter.gene.cor` parameter to use, I can say it's both awesome and mysterious. Awesome because camera's rankings in this mode are often very useful, but mysterious because: what do the p-values now mean, really? How much should the analyst care? The original camera paper goes to some length to discuss the importance of type I error control and that camera's approach of estimating and accounting for inter-gene correlation is an improvement there. Given that the user can now override it, what can the analyst reasonably say about type I error control, now? Some guidance on choice of the value, or (perhaps) comment on why it's not so critical could be useful.

**Minor Comments**

- The authors have done a good job of enabling easy reproducibility of this workflow. Keeping with that spirit, it might be useful to change the code that materializes the `targets` object to be executable without the use of an external file. Leveraging R's ability to read from a `textConnection` might be one day to do that without loosing readability of the workflow, since the targes file would also be printed to the document without having to output its value:
          targets <- read.delim(textConnection("
                     GEO        SRA CellType    Status
      MCL1.DG GSM1480297 SRR1552450        B    virgin
      MCL1.DH GSM1480298 SRR1552451        B    virgin
      MCL1.DI GSM1480299 SRR1552452        B  pregnant
      MCL1.DJ GSM1480300 SRR1552453        B  pregnant
      MCL1.DK GSM1480301 SRR1552454        B lactating
      MCL1.DL GSM1480302 SRR1552455        B lactating
      MCL1.LA GSM1480291 SRR1552444        L    virgin
      MCL1.LB GSM1480292 SRR1552445        L    virgin
      MCL1.LC GSM1480293 SRR1552446        L  pregnant
      MCL1.LD GSM1480294 SRR1552447        L  pregnant
      MCL1.LE GSM1480295 SRR1552448        L lactating
      MCL1.LF GSM1480296 SRR1552449        L lactating
      "), sep="")
- In the "Downloading the read counts" section, the authors say that the first column of the downloaded read counts is the total number of bases in exons or UTRs for the gene, but UTRs *are* exons (sorry, pet peeve of mine) -- perhaps "total number of of basepairs from coding and non-coding exons(?)"
- When construction the DGEList, why not show that you can now easily drop the `targets` data.frame into the DGEList's `$samples` slot like so:
     
  y <- DGEList(GenewiseCounts[,-1], group=group, samples=targets,
                   genes=GenewiseCounts[,1,drop=FALSE])

**Grammar / Spelling**

  

- In the "Filtering to remove low counts" section
      - 'Genes that have *with* very low counts across all the libraries ...'
      - As a rule of thumb, we require that gene have a count of ...'
          + fix this sentence in a few places to support the use of "gene" or "genes"
- After `plotBCV`: "The dispersion trend tends [to] decrease smoothly with abundance and (to -> is) asymptotic to a constant ..."
- When the concept of dispersion estimation is introduced, the different types of dispersions are briefly discussed (each individual gene, common dispersion, trended dispersion of a gene). Then, in the QL approach, you mention that the "tagwise NB dispersions" are not used. Would be useful to use same naming convention (ie. which of the previous types of dispersions introduced is the tagwise dispersion referenced here?)
- Remove third "the" here: "The QL functions moderte the genewise the QL dispersion estimates ..."
- Under *complicated contrasts*: Suppose we are interested in testing whether the change in expression between lactating and pregnant mice is the same for basal cells [as] it is for luminal cells.

I have read this submission. I believe that I have an appropriate level of expertise to confirm that it is of an acceptable scientific standard.

# Response

**Published:** 2016-07-22

**Authors:** Gordon Smyth

**Affiliations:** Walter and Eliza Hall Institute of Medical Research, Australia

Thanks, Steve, for your positive comments. Here are some responses to your extra suggestions.

***After the `plotMD` code example, the authors mention that "the bulk of genes should be centered at a line of zero log-fold change ...", it might be worth mentioning a few options to consider when a vanilla call to `calcNormFactors` doesn't produce that result.***

 A paragraph has been added at the end of the normalization section. The MD plots have been moved to the next section with some added discussion of the relationship to normalization factors.

***In the Introduction, the authors cite [12] when they mention edgeR's QLF framework offers some additional statistical refinements when compared to QuasiSeq ... Could the authors clarify what these refinements might be?***

 Robust empirical Bayes (robust=TRUE) is one refinement. Another is more careful treatment of zero counts when modelling the residual deviances. See our response to reviewer 1 (Conrad Burden).

***From previous experience of putting camera's `inter.gene.cor` parameter to use, I can say it's both awesome and mysterious. ... Some guidance on choice of the value, or (perhaps) comment on why it's not so critical could be useful.***

 See our response to reviewer 2 (Devon Ryan).

***The authors have done a good job of enabling easy reproducibility of this workflow. Keeping with that spirit, it might be useful to change the code that materializes the `targets` object to be executable without the use of an external file. Leveraging R's ability to read from a `textConnection` might be one day to do that without loosing readability of the workflow***

 That's a interesting suggestion. Alternatively of course we could have simply created the CellType and Status vectors in R without reading a targets frame at all. The reason why we read from an external file is we feel that doing so has pedagogic value. We think it is generally good practice for experimenters to create the targets file outside of R, using a spreadsheet editor like Excel. This forces the experimenter to check the correspondence between sample IDs and experimental factors. We want the workflow to mimic how a real analysis will go.

In the "Downloading the read counts" section, the authors say that the first column of the downloaded read counts is the total number of bases in exons or UTRs for the gene, but UTRs *are* exons

 We agree, but the text nevertheless seems simple and clear. In our experience, it helps to explicitly mention UTRs. We've changed it to "exons and UTRs".

***When constructing the DGEList, why not show that you can now easily drop the `targets` data.frame into the DGEList's `$samples` slot like so:***

 Good suggestion. We don't do it just so that the output from y$samples on pages 4 and 5 doesn't exceed the window width.

    

***Grammar / Spelling***

 Fixed.

***When the concept of dispersion estimation is introduced, the different types of dispersions are briefly discussed (each individual gene, common dispersion, trended dispersion of a gene). Then, in the QL approach, you mention that the "tagwise NB dispersions" are not used. Would be useful to use same naming convention (ie. which of the previous types of dispersions introduced is the tagwise dispersion referenced here?)***

 Good suggestion, done.

# Peer review: Referee response for version 1

**DOI:** 10.5256/f1000research.9667.r14480

**Published:** 2016-07-13

**Authors:** Tsung Fei Khang

**Affiliations:** 1 Institute of Mathematical Sciences, Faculty of Science, University of Malaya, Kuala Lumpur, Malaysia

First of all, please accept my apologies for not being able to complete the review earlier. I would like to thank the editor for the opportunity to review this interesting paper. EdgeR is one of the more popular methods for performing RNA-Seq data analysis, and the authors’ efforts in writing this expository tutorial will surely be welcome by students and researchers who need to work with analysis of RNA-Seq data.

 I think entry-level readers with basic R skills will find the workflow described easy to follow; and having actually worked out an example data set, will have less difficulty in adapting it to the needs of their own data analysis. However, intermediate or experienced readers might find the presentation of some parts of the workflow overly simplistic (as pointed out by one of the reviewers N.J.Schurch) - but this is not a weakness of the paper, since it is too much to expect that all the nuances of a refined RNA-Seq data analysis can be covered in this tutorial paper. Nonetheless, I believe the discussion of these points will potentially add value to the paper – they do not necessarily imply the necessity of revising the work to include the points raised, because the appending of reviewers’ comments enables readers to assess how relevant are the points raised to their own work.

 To ease discussion of the paper, I will itemize my comments as follows.

1. Despite declaring “From reads to genes to pathways” in their title, the authors choose not to develop the contents of their paper in this sequence. Rather, they start immediately with the count table, and then develop the material going from genes to pathways. The section on read mapping is presented at the end instead.
  I understand the focus of their sequence of writing, which is to get the readers into the heart of the action quickly, using the tool that they are most familiar with, edgeR. However, read processing is an important upstream checkpoint, and the things that one chooses to do at this stage has more important consequences than choices of which contrasts to make, optimizing plots, etc. Personally, I feel that insufficient attention has been given to this section, which can benefit from more discussion. It troubles me that there is no mention of short read quality control, a standard (and important) requirement for data quality check, which I am sure the authors are aware of. This is typically visualized using the standard tool FastQC. Subsequently, depending on the diagnostics, one can use a tool like FastX or Trimmomatic ^1^ to remove problematic segments, usually the 3' end. Then, there is a plethora of read mapping methods that can be used  (of which Rsubread is just one of them), and also methods of constructing the count table from the mapped reads. Optimal combinations of methods for performing both tasks were recently investigated by Fonseca *et al*. ^2^, who suggested combinations such as OSA+HTSeq for producing the reliable count tables. While the authors do not really need to show how these can be done, I think they should devote a short paragraph to discuss these issues because of their fundamental nature.
2. The authors demonstrate the use of mean difference (MD / a.k.a MA) plots as a diagnostic plot for checking data distributional properties. These are useful for checking whether variances increase as counts get larger in samples (the “fanning patterns”), for example. Less clear is the appropriate course of action in the event observing such undesirable patterns. Do we try to carry on the analysis, using log-transformed data? Do we discard problematic samples? Admittedly these are delicate issues that require more space for discussion than is possible in the paper. Nevertheless, providing some guidelines or pointing out useful references for further exploration will surely help readers appreciate the use of these plots.
3. There is strangely no illustration of how to make a volcano plot in the tutorial, which is a common graphical plot for assessing the joint relationship between statistical and biological significance. From experience, I find such plots important for understanding how different DE methods pick DE gene candidates.
  I was motivated to understand how *glmQLFTest *and *glmTreat *functions call DE genes compared to a simple method based on hyperbolic decision rules proposed by Xiao *et al.* ^3^ using the volcano plot. The method of Xiao constructs the decision rule as follows: Declare a fold change  (FC) cut-off below which one is not interested in a gene as a DE candidate. So if we desire FC > 2 for up-regulated genes (and conversely FC < 1/2 for down-regulated genes), then |log2(FC)| > 1. Next, we set the level of statistical significance, above which a gene is considered to be an unlikely DE candidate. Suppose we use the adjusted p-value, and require p < 0.01. This implies that –log10(p) > 2. If we denote y = -log10(p) and x = log2(FC), then the product of these two inequalities gives |x|y >  2, so that y > 2/|x|. This translates to a hyperbolic decision rule, such that genes with x and y values lying in the rejection region are selected as DE candidates. This rule allows one to include genes with very large FC but higher p-value. If we care a lot about managing false positives, then we could add a hard requirement for –log10(p) = 2, meaning that we will only considering genes that demonstrate adjusted p-values below 0.01.
  The result of my exploration is attached here. Non-DE genes are in black. The genes picked using *glmQLFTest *are in green; note the majority of them are also picked by the hyperbolic decision rule (blue). Candidates returned from *glmTreat* are boxed in purple, and seems to form a subset of the candidates returned from the hyperbolic decision rule. However, they show a peculiar distribution pattern, in that some genes with large log2(FC) and –log10(p) do not get picked. It is unclear to me why such genes are not detected by the algorithm. Regardless, a volcano plot is an important instrument that readers can have at their disposal for understanding the behaviour of DE gene calling algorithms.
4. The heatmap (Fig.7) is an important graphical plot of any gene expression analysis project, but there are some subtleties to its proper generation. I think it is not easy to explain the clustering pattern of the samples in Fig. 7, where basal and luminal cell samples are grouped together. Fortunately, this is often just a problem of the choice of clustering algorithm used. The default method in *heatmap.2* for clustering is complete linkage, which is often not the best method. From experience, changing it to the ward.D algorithm frequently produced biologically meaningful results, which is the case in the current analysis.
  The figure here shows a possible modification of the heatmap. Here, the basal and luminal samples nicely separate out into two clusters, following biological intuition. Columns of interest (e.g. lactating state in both basal and luminal cells) can be boxed to draw reader’s attention.
  May I also recommend that the srtCol argument in the heatmap be introduced to users, since sooner or later one would have to deal with space issues with labels on a heatmap, and what better way to handle this than having them oblique instead of perpendicular to the plot? Additionally, I think the outcome of customizing the heatmap using the given margin, lhei and lwid arguments will produce variable results in different computers (I got a "figure margins too large" error message initially), and so a note to users may be useful.
5. In the output table produced using the *topTags *function, there is a column named “FDR”. Should this be “Adjusted p-value”? In the Benjamini-Hochberg correction method that the authors’ used, FDR is a parameter determined by the user. Depending on one’s taste for false positive tolerance, one can tune it low or high (maybe useful to let users tune it?), so synonymizing “FDR” with “adjusted p-value” leads to conceptual confusion. By the way, how did the authors compute the adjusted p-value?
6. While testing out the codes, I noted that the output that I got differed slightly from those shown by the authors.  Additionally, like N.J.Schurch, I also encountered problems running the fry code example:
*> fry(y, index=cyt.go.genes, design=design, contrast=B.VvsL)*
*Error in array(x, c(length(x), 1L), if (!is.null(names(x))) list(names(x),  : 'data' must be of a vector type, was 'NULL'*
  Only much later in the end did I read, on page 21, that the authors made their analysis using R version 3.3.0 or higher, with Bioconductor version 3.3. Since my versions for both were 3.2, I suppose that the variation in output, as well as the error message seen, could be just a consequence of different versions. Would it be better if the versions used are announced right at the beginning of the paper? Additionally, it would also help if the packages needed for running the analysis are all installed at the beginning of the R script provided (e.g. readers who had not run *biocLite(“GO.db”)* to install the package from Bioconductor would get an error running *library(GO.db*) – this is not mentioned in the text I think, and can trouble beginners)
7. Minor comments:
  (i)Subject-verb agreement issue (page 6): “We require that gene(s) have a count ….”.(ii)ANOVDEV - analysis of deviance, a citation is useful. 

Note: Codes for producing the volcano plot and heatmap are available here.

I have read this submission. I believe that I have an appropriate level of expertise to confirm that it is of an acceptable scientific standard.

## References

[1] BolgerAMLohseMUsadelB: Trimmomatic: a flexible trimmer for Illumina sequence data. *Bioinformatics*.2014;30(15) : 10.1093/bioinformatics/btu170 2114-20 10.1093/bioinformatics/btu170 24695404PMC4103590

[2] FonsecaNAMarioniJBrazmaA: RNA-Seq gene profiling--a systematic empirical comparison. *PLoS One*.2014;9(9) : 10.1371/journal.pone.0107026 e107026 10.1371/journal.pone.0107026 25268973PMC4182317

[3] XiaoYHsiaoTHSureshUChenHIWuXWolfSEChenY: A novel significance score for gene selection and ranking. *Bioinformatics*.2014;30(6) : 10.1093/bioinformatics/btr671 801-7 10.1093/bioinformatics/btr671 22321699PMC3957066

# Response

**Published:** 2016-07-22

**Authors:** Gordon Smyth

**Affiliations:** Walter and Eliza Hall Institute of Medical Research, Australia

We thank the reviewer for his thoughtful comments, although we have different views on some of the specific issues raised.

 The reviewer is right that this is a tutorial aimed at entry-level readers. We went to some trouble to make the tutorial simple, but we do not think it is "simplistic". The article alludes to many data analysis issues in a concise style. It includes material that is new and interesting for an advanced reader, as the reviewer reports show.

 Our article is "live" in the sense that readers can regenerate the analysis and the article themselves automatically from a knitr file containing R code. In this revision, we have added a link to the code files. It isn't practical to include the read alignment in a live analysis because it is so much more computationally demanding than the rest of the analysis. Requiring readers to undertake the alignment step to obtain the read counts would drastically limit the audience who could go through the rest of the analysis.

 We have added a note to the article about plotting quality scores. However we think that trimming poor quality segments is an old-fashioned step that is generally unnecessary given improved sequencing protocols and high quality robust aligners like subread. Trimming is more likely to be harmful than helpful for a gene-level RNA-seq study. Indeed we think that encouraging entry-level users to make ad hoc edits of their sequence data is quite dangerous. It is far better to allow a quality-score-aware aligner like subread to make decisions on a per-read basis.

 We are not sure why the reviewer cites Fonseca et al (FMB), but we make the following points. FMB evaluated pipelines for quantifying absolute expression, which is not directly relevant in a differential expression study such as ours. FMB only compared pipelines available at the time. The OSA+HTSeq pipeline is not particularly popular, for example it has not been adopted by any of the FMB authors themselves for any published study of real data. By contrast, the Rsubread+featureCounts pipeline that we suggest is newer, faster and more widely used.

 We could cite references to claim superiority for the Rsubread and featureCounts tools, for example the SEQC study (Su et al, 2014), but a review of the literature would be out of place in our article. What is undoubtedly true is that Rsubread+featureCounts is more than good enough and easily the fastest and most convenient in an R context because of its native implementation as an R package.

 The reviewer may have misinterpreted the purpose of the MD plots. They are designed to display differential expression, either for individual samples or for a fitted model. They are not designed to check distributional properties. They do not check whether variances increase with count size. They are not used to suggest transformations of the data.

 Volcano plots were originally motivated by the shortcomings of ordinary t-tests, which can give very small p-values even for genes with tiny fold changes. However this problem has been overcome by empirical Bayes test statistics, and we do not generally recommend volcano plots in the context of an edgeR analysis. Volcano plots tend to encourage fold change cutoffs, which we also don't recommend. We much prefer the MD plot (Figure 5) because it shows clearly how larger fold changes are required to reliably call DE for lower expressed genes.

 The decision rule of Xiao et al (2014) doesn't give rigorous control of the FDR, and it has the tendency to prioritize genes with small counts that have large fold changes and large variances. We prefer not to prioritize lowly expressed genes. Again, this is made clear by the MD plot.

 Thank you for the suggestions and code for the heatmap. The pattern seen in our heatmap is because we chose to display genes that are DE between B.pregnant and B.lactating, hence it is natural that these two populations are separated at the far left and right of the plot. We agree that slanted labels are useful, as are some of the other options you demonstrate. The choice of clustering algorithm is controversial, especially so as the ward.D algorithm is not a correct representation of Ward's method, with some writers claiming that only ward.D2 should be used. Anyway, our article is not about heatmaps *per se*. Our aim is simply to provide an example of how results can be transferred to a heatmap, so it is best to keep the heatmap call as simple as possible. Users are then free to add as many embellishments as they like.

 There is no conceptual confusion with FDRs. For any FDR tolerance that a user might have, the genes for which the FDR value from topTags() is less than this cutoff are exactly the same genes that would be judged statistically significant by Benjamini-Hochberg's 1995 algorithm.

 Obviously the results from the workflow will differ slightly if older versions of R and Bioconductor are used. The software versions were stated on page 18 as well as on page 21. The journal format is that software requirements are described at the end of the article. The requirements seem to us to be well sign-posted in sections called "Packages used" and "Data and software availability". In any case, we are a bit surprised that readers should need special prompting to install the current versions of R and Bioconductor.

 Package installation is a "once off" operation, so we prefer not to make it part of the workflow code that a user might run many times.

 A citation for ANODEV has been added.

**Reference**

 Su, Z, et al (2014). A comprehensive assessment of RNA-seq accuracy, reproducibility and information content by the Sequencing Quality Control Consortium. Nature Biotechnology 32(9), 903-914.

# Peer review: Referee response for version 1

**DOI:** 10.5256/f1000research.9667.r14478

**Published:** 2016-07-06

**Authors:** Devon P. Ryan

**Affiliations:** 1 Max Planck Institute of Immunobiology and Epigenetics, Freiburg, Germany

RNAseq is easily one of the most prevalent NGS experiment types and edgeR one of the most heavily used tools for analyzing the results of these experiments. Given that, I'm quite pleased to see this article from Chen and colleagues that provides a very convenient walk-through of how to perform a typical analysis, including pathway and GO enrichment.

 I have no real reservations regarding this article. Below I'd like to point to a few parts of the paper that could use caveats or further explanation.

1. The example experiment has only two samples per group. That suffices in some circumstances, but at least in my experience the lay reader has the unfortunate habit of reading too much into the the number of samples used in papers like this and then trying to use that as justification for similar sample numbers for their much lower effect size experiments. A caveat or note of warning to those new to RNAseq would have been nice.
2. There's typically filtering done, such as the "rowSums(cpm(y) > 0.5) >= 2" in this paper. It would have been nice to include some recommendations regarding how to choose a filtering threshold.
3. The p-values produced by goana() and topKEGG() are presumably unadjusted for multiple testing. It would have been nice if there had been a note to not then use the typical 0.05 cut off.
4. While playing around with the code presented in the paper, I noticed that the choice of 0.01 for the "inter.gene.cor" parameter in camera() has a drastic affect on the resulting p-values. It would be incredibly useful to know when one should override the default value and how one should then derive an appropriate value. My concern is primarily that many will see these commands as "the one true method" for performing such an analysis and blindly apply the option in cases where it might not be appropriate.

I have read this submission. I believe that I have an appropriate level of expertise to confirm that it is of an acceptable scientific standard.

# Response

**Published:** 2016-07-22

**Authors:** Gordon Smyth

**Affiliations:** Walter and Eliza Hall Institute of Medical Research, Australia

Thank you for your thoughtful report.

 We have added a note about small sample numbers to the end of the section describing the experiment.

 To be honest, we thought that we had already explained how to choose the filtering threshold in some detail. Admittedly we explained in words how the 0.5 and the 2 values in the filter formula were derived, and why they are appropriate for this experiment, rather than giving a formula. Anyway, we have edited the filtering section and added a couple of sentences.

 We have added a note about the p-values from topGO() and topKEGG(). We generally ignore p-values above about 1e-5.

 You ask a good question about inter.gene.cor for camera(). We have recently made inter.gene.cor=0.01 the default setting for camera(). Previously the default was to estimate the correlation separately for each gene set. The old default gives rigorous control of the type I error rate but is conservative and doesn't always rank the most biological interpretable sets most highly. The ranking issues occurs because of the need to penalize highly co-regulated sets with positive inter-gene correlations. We and others (Tarca et al, 2013) have noticed that much simpler methods like limma's geneSetTest() tend to give a better ranking of the biologically significant sets although they do not control the error rate correctly. Our recent use of a preset value for inter.gene.cor in camera() is an attempt to strike a compromise between the original camera() and geneSetTest(). Note the latter is equivalent to camera() with inter.gene.cor=0. The compromise gains the advantages of geneSetTest() while keeping reasonable, although not perfect, error rate control. You are right that the camera p-values are sensitive to the value for inter.gene.cor. Nevertheless, after quite a bit of experimentation, we have chosen the value of 0.01 as a reasonable compromise between ranking and error rate control that gives good results across a range of datasets. So we are happy to offer it for general use and would prefer that most users kept to the default value. The p-values will often be somewhat optimistic, but probably not more so than other commonly used methods like the Fisher tests for GO and KEGG terms. It gives vastly better error control than gene sets methods that permute genes and ignore inter-gene correlations, especially for larger sets.

**Reference**

 Tarca AL, Bhatti G, Romero R (2013) A comparison of gene set analysis methods in terms of sensitivity, prioritization and specificity. PLoS ONE 8(11): e79217. http://dx.doi.org/10.1371/journal.pone.0079217

# Peer review: Referee response for version 1

**DOI:** 10.5256/f1000research.9667.r14475

**Published:** 2016-07-01

**Authors:** Nicholas J. Schurch

**Affiliations:** 1 Division of Computational Biology, College of Life Sciences, University of Dundee, Dundee, UK

This paper lays out a clear and relatively concise example of a linear workflow for analysing an RNA-seq based Differential Gene Expression experiment. The workflow focuses on doing all the analysis steps within R using the author's preferred tools (Rsubread & edgeR) and extends usefully to common aspects of pathway analysis with GO terms, Kegg terms and Gene Set testing.

 While I'm sure this paper will be useful for a section of the community (particularly newcomers to this kind of analysis), I find the paper to be quite simplistic and lacking in depth and discussion. In particular, there is no discussion of the subtleties involved in performing this kind of analysis at the coalface of scientific research.

 Some particular points I would like to have seen discussed are:

1. How many replicates should be used for this kind of analysis.
  The example study uses a very poorly replicated dataset. In this case two replicates per condition *may* be sufficient, but not only is it not discussed but for most experiments this is highly misleading! For new RNA-seq experiments a significantly higher number of replicates should be used, both to guard against problem samples/libraries and to ensure sufficient statistical power to identify significant differential expression (in particular because it is rare to know how large the changes in the data will be before you do the experiment).
2. How might one identify problematic issues with datasets that aren't as cooperative as the example dataset.
  For example, what would significant structure or curvature in the point cloud of the MD plot signify? If the samples don't cluster nicely on the MDS plot by condition, what might this mean (mislabelling, bad replicates, etc)?
3. How one might remedy or deal with the problems in such uncooperative datasets.
  For example, what general approaches could be used for isolating the root cause of the observed problems? How might we adjust the analysis to ameliorate the problems and their downstream impact (dropping datasets, changing mapping parameters, filtering the data)?
4. How one might go about choosing sensible selections and thresholds for the data.
  In my opinion the use of 'standard' and/or 'default' parameters, thresholds and selections (e.g. unique=TRUE, FDR>0.05, log2(FC)>1, cpm>0.5, etc) is a significant and endemic problem in this field. Often these are used solely because they have been used widely before, rather than considering whether they are appropriate for the specific data being analysed. What caused the authors to choose the values they use for this data and what key plots or pieces of information are valuable for choosing these appropriately?
5. How the various selection steps, thresholds and even the version of the software used, might impact on the downstream results.
  For example, if you change the FDR threshold from 0.05 to 0.01, how does this impact the downstream pathway analysis? Are some of these analyses insensitive to threshold values (e.g. gene set analysis) and does this make them better/more useful? If you change the cpm threshold to 1.0, or if you allow non-unique read mappings, how does this impact the number of identified SDE genes and the downstream pathway analyses?

I am not suggesting that the authors should have given an exhaustive account of the issues. Rather, I think the paper would benefit from briefly discussing some of the more subtle and complex issues surrounding these types of analyses and perhaps highlight some key problems, parameters and thresholds that should be thought about carefully. Without this the paper really presents a very linear, idealized, example of what, in practice, are complex analyses that may require considerably more thought and investigation.

 I also encountered some more specific issues:

1. The link provided does not (currently) link to the actual bioconductor workflow.
2. I ran all the R commands and they all work (except 'fry' see point 4 below), however I didn't get exactly the same results when (and after) filtering out genes without a symbol.
  The paper has:
*> head(y$genes)*
*                   Length  Symbol*
*497097          3634     Xkr4*
*  100503874    3259     Gm19938*
*  100038431    1634     Gm10568*
*19888            9747     Rp1*
*20671            3130     Sox17*
*27395            4203     Mrpl15*
*> y <- y[!is.na(y$genes$Symbol), ]*
*> dim(y)*
*[1] 26357    12*
  I had:
*> head(y$genes)*
*                 Length  Symbol*
*497097         3634     Xkr4*
*  100503874   3259     Gm19938*
*  100038431   1634     Gm10568*
*19888           9747     Rp1*
*20671           3130     Sox17*
*27395           4203     Mrpl15*
*> y <- y[!is.na(y$genes$Symbol), ]*
*> dim(y)*
*[1] 26608    12*
  The change is relatively small but it cascades causing differences in the genes that pass cpm filtering, differences in the normalization factors and differences in the DE results and the downstream analyses. I suspect this is the result of using a slightly older version of org.Mm.eg.db (3.2.3, vs 3.3.0) due to using an older version of R (3.2 vs 3.3). This goes nicely to point (5) above.
3. There is no real description of the reasoning behind scaling of the heatmap values to a mean of zero and std dev of one.
4. The 'fry' command failed for me, producing the error:
*> fry(y, index=cyt.go.genes, design=design, contrast=B.VvsL)*
*Error in array(x, c(length(x), 1L), if (!is.null(names(x))) list(names(x),  :*
* 'data' must be of a vector type, was 'NULL'*

I have read this submission. I believe that I have an appropriate level of expertise to confirm that it is of an acceptable scientific standard.

# Response

**Published:** 2016-07-22

**Authors:** Gordon Smyth

**Affiliations:** Walter and Eliza Hall Institute of Medical Research, Australia

We thank the reviewer for approving our article.

 We also thank the reviewer for his comments about RNA-seq analysis in general, but we didn't find the suggestions for revisions to be helpful. One problem is that the reviewer did not follow the instructions given in the article regarding software requirements. In general, Bioconductor workflows and Bioconductor channel articles are intended to be run using the latest release of Bioconductor. This was explicitly explained in the article, which said "This workflow depends on various packages from version 3.3 of the Bioconductor project, running on R version 3.3.0 or higher." The article went on to give the version numbers of all packages used. Unfortunately the reviewer tested the workflow using an earlier version of Bioconductor, with the result that one of the function calls didn't work and there were slight changes in the annotation.

 We make no apology for providing a "linear" workflow demonstrating an easy, robust, fast and flexible workflow from RNA-seq reads through to pathway analysis. That was clearly the purpose of the article. We are pleased that the reviewer found our analysis to be "idealized" because it is in fact a typical example of our own biomedical research that we published recently in Nature Cell Biology. The article gives an insight, as far as is possible in a short journal article, of our own analysis process "at the coalface of scientific research".

 The reviewer wants us to take lots of sidepaths, discussing problems that did not in fact occur, but we think that readers will not want to be distracted by hypothetical dead-ends in this way. The instructions for writing these articles (available from https://support.bioconductor.org/p/80077) advised authors to take a pragmatic task-orientated approach and not to get bogged down with extensive discussions of options. One has to start with an example of how an analysis should work in order to have a firm basis from which to deal with problematic studies that might arise in the future.

 The reviewer also wants us to discuss the consequences of myriad perturbations of thresholds and parameters in the analysis pipeline. On one hand, we are disappointed that the reviewer failed to acknowledge the many explanations that were given in the article. On the other, we think that the specific parameter settings questioned by the reviewer are not particularly crucial and are not the most important issues that we would like researchers to be thinking about when they conduct an analysis.

 Regarding sample sizes, the minimum appropriate sample size depends very much on the context. n=2 may be sufficient when comparing well sorted cell types from genetically identical mice whereas n=10 may be not nearly enough when comparing whole blood from diseased vs normal patients. For the study analyzed in our article, the cell types have distinct and highly reproducible expression profiles. The results were validated in the biological publication (Fu et al, 2015) in a number of ways. The repeatability of the results was also demonstrated in our current article by the strong correlation between the current results and earlier microarray results on similar cell populations (Figure 9). We disagree with the reviewer's position that sample size recommendations can be made independently of the biological context and the purpose of the scientific study.

 We now give responses to specific issues raised by the reviewer:

***1. How many replicates should be used for this kind of analysis.***

 This is not an article about experimental design.

***2. How might one identify problematic issues with datasets that aren't as cooperative as the example dataset.***

 The article already shows users how to create appropriate plots from which problems can be identified.

***3. How one might remedy or deal with the problems in such uncooperative datasets.***

 Trying to give a solution to every possible problem that might arise is clearly beyond the scope of the current article. In many cases it might be that no special action needs to be taken as the pipeline we give is quite robust.

***4. How one might go about choosing sensible selections and thresholds for the data. What caused the authors to choose the values they use for this data and what key plots or pieces of information are valuable for choosing these appropriately?***

 This has already been explained where relevant in the article.

***In my opinion the use of 'standard' and/or 'default' parameters, thresholds and selections (e.g. unique=TRUE, FDR>0.05, log2(FC)>1, cpm>0.5, etc) is a significant and endemic problem in this field. Often these are used solely because they have been used widely before, rather than considering whether they are appropriate for the specific data being analysed.***

 The reviewer is entitled to his point of view about the field in general, but all these issues are addressed from first principles in our article.

 The reason for setting unique=TRUE in the call to align() was explained at the top of page 23. We see no point in unique=FALSE for a gene-level expression analysis.

 We did not use a 'standard' cpm cutoff but rather explained how to work out a useful threshold for this specific data set. In fact the pipeline is robust to the filtering and tends to give similar results for a range of filtering methods and thresholds, as explained in the article.

 We provided an extended discussion of DE cutoffs on pages 12-13 (nearly two whole pages). We presented a sophisticated solution using glmTreat() that is better than using a FC cutoff or making the FDR cutoff more stringent.

***5. How the various selection steps, thresholds and even the version of the software used, might impact on the downstream results.***

 Already explained where appropriate. For example, page 18 says "Gene set tests consider all the genes in the specified set and do not depend on any pre-emptive significance cutoff."

 In most cases, changes to the thresholds either have obvious effects (a lower FDR cutoff produces fewer DE genes) or have less impact than the reviewer seems to imply.

***1. The link provided does not (currently) link to the actual bioconductor workflow.***

 We submitted our workflow to Bioconductor at about the same time as submitting to F1000Research but it has not yet appeared on the Bioconductor website. Unfortunately, none of the Bioconductor channel articles on F1000Research link to a corresponding code workflow.

 In the meantime, we have made our code and data available from http://bioinf.wehi.edu.au/edgeR/F1000Research2016 and have added this link to the revised article. Note that the entire LaTeX article is generated automatically by running knit() on a Rnw file.

***2. I ran all the R commands and they all work (except 'fry' see point 4 below), however I didn't get exactly the same results when (and after) filtering out genes without a symbol.***

 You used out-of-date versions of R and Bioconductor.

***The change is relatively small but it cascades causing differences in the genes that pass cpm filtering, differences in the normalization factors and differences in the DE results and the downstream analyses.***

 In fact the DE results are almost identical using either Bioconductor 3.2 or 3.3, demonstrating the robustness of the pipeline.

***3. There is no real description of the reasoning behind scaling of the heatmap values to a mean of zero and std dev of one.***

 The rationale was explained (it makes Euclidean distance a function of correlation). We have added one more sentence to the revised article.

***4. The 'fry' command failed for me, producing the error:***

 There was no DGEList method for fry in the Bioconductor 3.2.

# Peer review: Referee response for version 1

**DOI:** 10.5256/f1000research.9667.r14473

**Published:** 2016-06-29

**Authors:** Conrad J. Burden

**Affiliations:** 1 Mathematical Sciences Institute, Australian National University, Canberra, ACT, Australia; 2 Research School of Biology, Australian National University, Canberra, ACT, Australia

There are two main contenders for off-the-shelf software packages for detecting differential expression from RNA-Seq count data: edge R and DESeq2, both of which model over-dispersed count data with a negative binomial distribution. A complete work flow built around DESeq2 was published recently ^1^, and this is the analogous complete work flow, starting from raw sequence counts, for edgeR. The example given goes further in that it also includes an analysis right through to a molecular pathway analysis of the most highly differentially expressed genes.  

 The work flow is built around a number of edgeR functions which have been developed and improved over years. The most recent development is the inclusion of a method employed in an earlier package, called QuasiSeq, which combines a quasi-likelihood approach to estimating estimating over-dispersion with edgeR’s traditional approach of sharing information across genes. In my own review of packages designed for profiling differential expression from count data ^2^ (cited as ref. 11 in this paper) I observed using synthetic data that QuasiSeq easily outperformed the then existing available packages in terms of accuracy of claimed p-values and false discovery rates. However, it did have the disadvantage of very poor performance in terms of speed. The functions glmQLFit() and glmQLFtest() in the current work flow perform the quasi-likelihood method, but have overcome the speed performance problem completely and run very rapidly. Incidentally I can confirm that I and my co-authors of ^2^ have no particular connection with either the edgeR or DESeq groups, or the developers of QuasiSeq.  

 One problem I have with the example used in the workflow is that there are only two biological replicates in each condition. In ref. ^2^ we observed using synthetic data that to get consistently accurate estimates of p-values and false discovery rates it is best to use at least 3 and preferably 4 biological replicates in each condition, even with QuasiSeq. See also the recent review by Schurch *et al.* ^3^ whose analysis recommends far more than two biological replicates in general with various software packages available at the time of their analysis. A similar independent analysis of the number of biological replicates recommended for the latest edgeR work flow would be welcome.  

 I have worked through the example given in the paper, starting with “Downloading the read counts” on page 4, working through to pathway analysis ending on page 20. I have not worked through the “Read alignment and quantification” section starting on page 22. In general I found the work flow easy to follow and informative.  I have made the following observations: 

1. When I got to the line 
  y <- DGEList(…, group=group, …)
  on page 5, the parameter ‘group’ had not been set. I had to do a bit of detective work and, as a workaround, set it up using the following lines of code: 
      
  CellType <- c(rep("B", 6), rep("L", 6))
  Status <- rep(c(rep("virgin", 2), rep("pregnant", 2), rep("lactating", 2)),2)
  group <- paste(CellType, Status, sep=".")
  group <- factor(group)
  This should be fixed.
2. Suggestion: For ease of use, could calculating the TMM normalisation factors be built into the function DGEList()? If culling the low-count genes makes a noticeable difference, perhaps this could be done just as easily to the original data frame of counts before applying DGEList().
3. Regarding the diagnostic plot Figure 1, can it happen that the TMM normalisation doesn’t give an MD plot which is symmetric about zero? And if it does, is there a fix?
4. As a future enhancement, could a more user-friendly version of the differential expression analysis be made with estimateDisp(), glmQLFit() and glmQLFtest() all built into a single function? The point is that the job of the first two functions, i.e. calculating the trend dispersion and GLM coefficients, can’t be avoided anyway if you are a biologist wanting to do a differential expression analysis. For many users who are not familiar with the negative binomial model, the diagnostic plots of the BCV (Figure 3) and QL dispersion (Figure 4) are likely to be too arcane to be helpful. In fact the tagwise dispersions in Figure 3 are not actually used by the QL method.
5. A couple of trivial typos: 
  Page 5, 4th last line: “Genes that have with very low counts …”, remove “with”.
  Caption to Figure 4: “trend show in Figure effig:plotBCV” should be “trend shown in Fig.3”
  Page 16 last line: “B.pregant” should be B.pregnant”.

I have read this submission. I believe that I have an appropriate level of expertise to confirm that it is of an acceptable scientific standard.

## References

[1] LoveMIAndersSKimVHuberW: RNA-Seq workflow: gene-level exploratory analysis and differential expression. *F1000Res*.2015;4: 10.12688/f1000research.7035.1 1070 10.12688/f1000research.7035.1 26674615PMC4670015

[2] BurdenCJQureshiSEWilsonSR: Error estimates for the analysis of differential expression from RNA-seq count data. *PeerJ*.2014;2: 10.7717/peerj.576 e576 10.7717/peerj.576 25337456PMC4179614

[3] SchurchNJSchofieldPGierlińskiMColeCSherstnevASinghVWrobelNGharbiKSimpsonGGOwen-HughesTBlaxterMBartonGJ: How many biological replicates are needed in an RNA-seq experiment and which differential expression tool should you use?. *RNA*.2016;22(6) : 10.1261/rna.053959.115 839-51 10.1261/rna.053959.115 27022035PMC4878611

# Response

**Published:** 2016-07-22

**Authors:** Gordon Smyth

**Affiliations:** Walter and Eliza Hall Institute of Medical Research, Australia

Dear Conrad,

 Thank you for your thoughtful review and for your positive remarks about the performance of the quasi-likelihood pipeline.

 We understand that the code as given in the article would not run for you completely without the 'targets.txt' file that is read in the first code line. The whole LaTeX article and all the results are actually generated from a knitr Rnw file. The code and associated files have been submitted to Bioconductor as a workflow but are not yet available from the Bioconductor website. In the meantime, we have made the code and associated files available from our own website at http://bioinf.wehi.edu.au/edgeR/F1000Research2016.

 We find TMM normalization works well for almost all regular gene expression studies. Different normalization methods are more appropriate for other technologies that yield a lot of zeros, for example single-cell RNA-seq, CRISPR, ChIP-seq and Hi-C. Discussion of those is beyond the scope of this article but we have added a couple of references. Note that the MD plot in Figure 1 does not need to be symmetric, as long as the majority of points cluster around the line, and the article now clarifies this point.

 Our preference is to provide a modular pipeline, encouraging analysts to examine the results at each step. For example, we can't decide whether filtering or TMM normalization is appropriate for a particular study at the time of forming the DGEList. We also like to encourage users to examine the BCV plot. We find this an informative diagnostic plot even as part of limma pipelines that do use the estimateDisp() results. On the other hand, if the number of samples was very large, one might choose to compute just the NB dispersion trend and not the tagwise values.

 Thanks for pointing out the typos. They will be fixed in the next revision.

 Regarding sample sizes, the minimum appropriate sample size depends very much on the context. For example, n=2 may be sufficient when comparing well sorted cell types from genetically identical mice whereas n=10 may not be not nearly enough when comparing whole blood for diseased vs normal patients. The repeatability of the results in our workflow is demonstrated by, among other things, the strong correlation between the current results and earlier microarray results on similar cell populations (Figure 9).

 For cutting edge biomedical experiments, RNA samples can be very difficult to obtain. While larger sample sizes are always preferable, our philosophy is to perform the best possible data analysis for any experiment that our colleagues believe is scientifically worthwhile. It is our aim that edgeR-QL and limma should give statistically correct results for any sample size, even down to n=2 vs n=1. On this topic, we note that the current edgeR-QL code is more robust than the original QuasiSeq method when the sample sizes are very small. Note that QuasiSeq was based on our best understanding of the mathematics at the time of Lund et al (2012), but some important refinements have been added to the edgeR version since. Here is a very small simulated example with n=2 vs n=1 and no true differential expression. Here QuasiSeq gives FDR values as small as 0 or 0.01, whereas the smallest FDR from glmQLFTest is 0.97:

 > y <- matrix(rpois(10000*3,lambda=10),10000,3)

 > library(QuasiSeq)

 > design0 <- matrix(1,3,1)

 > design1 <- cbind(1,c(0,1,1))

 > design.list<-vector("list",2)

 > design.list[[1]] <- design1

 > design.list[[2]] <- design0

 > fit <- QL.fit(y, design.list)

 > res <- QL.results(fit)

 > lapply(res$Q.values, min)

 $QL

 [1] 0

 $QLShrink

 [1] 0.0138122

 $QLSpline

 [1] 0.01351794

 > library(edgeR)

 Loading required package: limma

 > dge <- DGEList(counts=y)

 > dge <- estimateDisp(dge, design1)

 > fit <- glmQLFit(dge, design1)

 > ql <- glmQLFTest(fit)

 > topTags(ql)

 Coefficient:   

          logFC   logCPM        F      PValue       FDR

 7645 -1.911573 6.981129 42.64028 0.008218779 0.9733061

 4209  3.366857 6.640090 41.30839 0.008583583 0.9733061

 4669  2.451686 7.159465 35.31776 0.010624873 0.9733061

 8608 -1.645226 6.942654 34.64701 0.010904508 0.9733061

 8402  2.224008 6.981127 33.15403 0.011573724 0.9733061

 2152  2.173823 6.942653 32.70039 0.011790966 0.9733061

 6240  2.568286 6.777594 32.50399 0.011887171 0.9733061

 7053 -1.743023 6.777595 32.50192 0.011888190 0.9733061

 6984  2.780434 6.942653 32.01302 0.012133576 0.9733061

 6057 -1.964729 6.488089 30.77110 0.012797066 0.9733061
